# Traditional Chinese medicine for the treatment of cancers of hepatobiliary system: from clinical evidence to drug discovery

**DOI:** 10.1186/s12943-024-02136-2

**Published:** 2024-10-01

**Authors:** Junyu Wu, Guoyi Tang, Chien-Shan Cheng, Ranna Yeerken, Yau-Tuen Chan, Zhiwen Fu, Yi-Chao Zheng, Yibin Feng, Ning Wang

**Affiliations:** 1 School of Chinese Medicine, the University of Hong Kong, 3, Sasson Road, Pokfulam, Hong Kong; 2 Department of Digestive Endoscopy Center & Gastroenterology, Shuguang Hospital Affiliated With Shanghai University of Traditional Chinese Medicine, Shanghai, 201203 China; 3 Department of Traditional Chinese Medicine, Shanghai Jiao Tong University School of Medicine Affiliated Ruijin Hospital, Shanghai, China; 4 Department of Pharmacy, Union Hospital, Tongji Medical College, Huazhong University of Science and Technology, Wuhan, China; 5 State Key Laboratory of Esophageal Cancer Prevention &, Treatment Institute of Drug Discovery and Development, School of Pharmaceutical Sciences, Zhengzhou University, 100 Kexue Avenue, Zhengzhou, Henan 450001 China

**Keywords:** Chinese medicine, Liver cancer, Biliary cancer, Pancreatic cancer, Clinical trials, Drug discovery

## Abstract

Hepatic, biliary, and pancreatic cancer pose significant challenges in the field of digestive system diseases due to their highly malignant nature. Traditional Chinese medicine (TCM) has gained attention as a potential therapeutic approach with long-standing use in China and well-recognized clinical benefits. In this review, we systematically summarized the clinical applications of TCM that have shown promising results in clinical trials in treating hepatic, biliary, and pancreatic cancer. We highlighted several commonly used TCM therapeutics with validated efficacy through rigorous clinical trials, including Huaier Granule, Huachansu, and Icaritin. The active compounds and their potential targets have been thoroughly elucidated to offer valuable insights into the potential of TCM for anti-cancer drug discovery. We emphasized the importance of further research to bridge the gap between TCM and modern oncology, facilitating the development of evidence-based TCM treatment for these challenging malignancies.

**Graphical Abstract:**

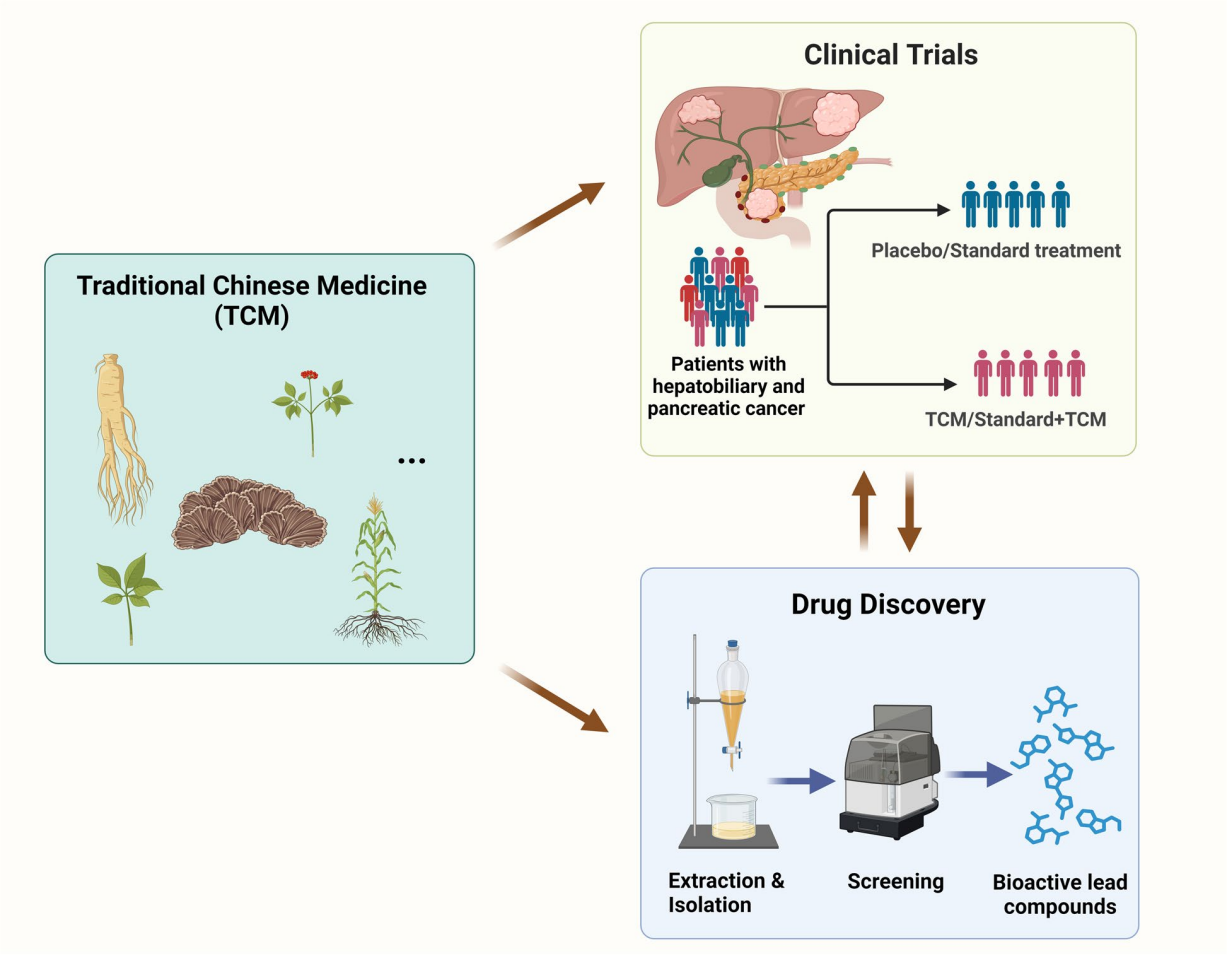

**Supplementary Information:**

The online version contains supplementary material available at 10.1186/s12943-024-02136-2.

## Introduction

Hepatic, biliary, and pancreatic cancers pose substantial clinical challenges in current medical practice. These malignancies are often diagnosed at advanced stages, leading to limited treatment options and poor prognoses. The unmet clinical needs in these cancers include several crucial areas, such as the development of effective early detection methods, implementation of personalized treatment strategies, overcoming therapy resistance mechanisms and enhancing palliative care approaches [1–4]. Addressing these challenges would undoubtedly bring significant benefit to improve patient outcomes and enhance the overall quality of care in the management of these malignancies. Traditional Chinese medicine (TCM), rooted in a holistic and individualized approach, offers a promising avenue for cancer treatment [5]. With a rich history of use, TCM offers a vast repertoire of plant-based compounds that exhibit diverse pharmacological activities [6, 7]. These compounds, often used in synergistic combinations within traditional formula, have shown favorable anti-cancer effects and bring inspirations to drug discovery as lead compounds [8, 9]. Numerous clinical studies have demonstrated beneficial outcomes, including improved tumor response, enhanced quality of life, and prolonged survival rates among cancer patients treated with Chinese medicine interventions, underlining the potential of TCM based cancer therapeutic approaches.

In this review, we aim to provide a comprehensive overview of the current clinical and scientific evidence regarding the application of TCM in hepatic, biliary, and pancreatic cancer treatment. According to pyramid of medical evidence, the majority of clinical study summarized in this review represent the highest levels of clinical evidence including randomized clinical trials (RCTs) and network meta-analysis (NMA). We included RCTs with a minimum sample size exceeding 30 participants and meta-analysis comprising data from at least 9 RCTs, with TCM as single or combinational agents for hepatobiliary system cancer treatment. Additionally, three single-arm phase I/II trials were incorporated to evaluate the biosafety profile of TCM. We highlighted the TCM formula that have been substantiated by robust clinical evidence, including large-population multicenter RCTs and NMA. By elucidating the underlying bioactive compounds inherent to these formulas, we aim to identify the potential lead compounds and their potential targets as well as pharmacological mechanisms. Additionally, we discussed the challenges and future directions in the clinical integration of TCM for cancer management, highlighting the need for further research, standardization, and collaboration between TCM and modern oncology.

## Clinical evidence and application of traditional Chinese medicine for the treatment of hepatic, biliary, and pancreatic *cancer*

### Hepatic *cancer*

Hepatic cancer is a significant global health burden, accounting for a substantial number of cancer-related deaths worldwide [3]. Hepatocellular carcinoma (HCC) is the most common type of primary hepatic cancer [10]. For patients with early stage of HCC, surgical resection is the most commonly used therapeutic approach, with 5-year survival of 38% – 61% [11–13]. However, postoperative recurrence remains a formidable challenge that significantly impacts the prognosis of patients who undergo hepatectomy. The 5-year recurrence rate can reach remarkably high levels, ranging from 50 to 80% [14–16]. For intermediate-stage HCC, transarterial chemoembolization (TACE) is a widely employed therapeutic approach. This procedure has shown favorable outcomes in terms of tumor size reduction, offering a potential bridge to curative therapies such as hepatectomy [17]. The development of treatment resistance and side effects like liver ischemia restrain its efficacy and the prognosis of patients [18]. Of note, fairly large proportion of HCC patients are diagnosed at advanced stages. In such cases, HCC is unresectable and only systemic therapy are available. To date, sorafenib and lenvatinib, two multi-kinase inhibitors, remain the first-line therapeutic agents for advanced HCC, showing significant efficacy in decrease disease progression [19, 20]. However, drug resistance persists as a challenging issue that hampers the sustained clinical benefits of tyrosine kinase inhibitors (TKIs) [21–23]. In recent years, immune checkpoint inhibitors have emerged as a breakthrough in HCC treatment. Nivolumab and pembrolizumab, both immune checkpoint inhibitors targeting programmed cell death protein 1 (PD-1), have also been approved as first-line treatments for advanced HCC. Despite notable advancements, durable clinical benefit remains limited to a small subset of patients, underlining the significant therapeutic challenges that persist [24, 25].

TCM possesses a diverse array of bioactive compounds that exhibit multi-faceted pharmacological activities, including anti-inflammatory, anti-angiogenic, and immune-modulatory effects. The multi-component nature enables targeting of multiple pathways involved in HCC development and allow personalized treatment approaches based on individual patterns of disharmony. Indeed, TCM could bring clinical benefits in HCC patients and holds potential in solving unmet medical needs. Liu et al. reported a controlled clinical trial in which 3483 patients with HCC were enrolled in. It was demonstrated that long-term use of TCM could bring survival benefit and was an independent protective factor for 5-year survival (adjusted HR = 0.46, 95% CI: 0.40 – 0.52,* p* < 0.0001) [26]. Besides, the multi-target property and favorable biocompatibility makes TCM a viable option for combining with chemotherapy to enhance treatment outcomes while reducing side effects [27–29]. Previous systemic meta-analysis reported that TCM could efficiently alleviate chemotherapy-induced peripheral neuropathy, leukopenia, and myelosuppression [29–31]. Moreover, a meta-analysis conducted by Xu et al. showed that TCM effectively enhanced the efficacy of TACE (OR = 1.88, 95% CI: 1.34 – 2.64, *p* = 0.03) [32]. We summarized TCM with solid clinical evidence as independent therapeutic agents or complementary approaches in combination with standard HCC treatment, including Huaier Granule, Jinlong Capsule, Compound Kushen Injection, Ginsenosides, Huachansu Tablet, Kangai Injection, Kanglaite Injection, Jianpi Huayu Therapy, Jiedu Granule, Biejia Ruangan Compound, and PHY906. TCM show improved response rates, prolonged progression-free survival, enhanced quality of life, and synergistic effects when combined with convention therapy in patients with HCC.

As mentioned before, the postoperative recurrence of HCC significantly dampens the prognosis of patients who receive primary HCC resection [33, 34]. Sorafenib, the first-in-line systemic therapy for primary HCC, however, failed to provide postoperative survival benefits in patients who underwent tumor resection according to a phase III STORM trial [35]. Unfortunately, there is currently a lack of approved adjuvant systemic therapeutic agents for the management of postoperative HCC recurrence [36, 37]. Of note, TCM has garnered significant attention as a potential postoperative adjuvant therapeutic approach due to its immunomodulatory effects and synergistic effects with conventional therapy. Huaier Granule is a TCM approved by Chinese National Medical Products Administration (NMPA) to be used alone or in combination with conventional therapy for the treatment of various cancer [26, 38]. Huaier Granule has been reported to bring significant clinical benefits in prolonging overall survival (OS) rates in HCC patients [39]. In 2018, a multi-center, randomized, parallel-controlled, phase IV clinical trial was conducted to evaluate the therapeutic potential Huaier Granule in preventing postoperative HCC recurrence [40]. Totally 1044 patients from 39 hospitals who underwent curative HCC resection were recruited and randomized in 2:1 ratio to receive either Huaier Granule or no additional treatment for up to 96 weeks. The Huaier group demonstrated a significantly higher RFS rate compared to the control group (62.39% *vs*. 49.05%, *p* = 0.0001). A significantly lower recurrence rate was also noted in Huaier group (37.61% *vs*. 50.96%, *p* = 0.0001). The effects of Huaier in preventing recurrence led to higher 96-week OS rates in the Huaier group compared to the control group (95.19% *vs*. 91.46%, *p* = 0.0207). The adverse effects were reported as mild and tolerable, suggesting that Huaier as TCM holds good biosafety and biocompatibility as a postoperative systemic therapy. This phase IV trial is the first nationwide multicenter study, which shed light on the therapeutic potential of TCM in addressing unresolved clinical dilemmas. One limitation of this study is the lack of placebo control, which also appears in many other clinical trials of TCM. This is owed to the distinctive taste of Huaier granule, making it quite difficult to develop a reliable placebo. Likewise, a cohort study incorporating 340 HCC patients with thermal ablation also demonstrated that postoperative treatment of Huaier significantly promoted OS rates and decreased the probability of recurrence (HR = 0.67, 95%CI: 0.49 – 0.93, *p* = 0.015) [41]. They suggested that continuous administration of Huaier over 2 years brought even better efficacy. Nevertheless, the cohort study's evidence level and sample size are suboptimal, emphasizing the necessity for additional large multicenter randomized controlled trials to substantiate the clinical efficacy of Huaier granule in the treatment of HCC.

Additionally, TCM are commonly used in combination with TACE, which suffers from incomplete tumor response, tumor recurrence, and adverse effects [18, 42]. According to a randomized, double-blind, placebo-controlled trial, Jianpi Liqi Decoction, which is a commonly used TCM with liver protective function, could significantly alleviate postembolization syndrome such as fever, pain, lack of appetite, drowsiness, dry mouth, and constipation (*p* < 0.05) [43]. Additionally, TCM is widely used in combination with TACE for synergistic therapeutic effects [44, 45]. Radix Ginseng is a famous TCM that has been widely used in clinical cancer management with well-documented anti-cancer activity and immune regulatory properties [46–48]. In a meta-analysis incorporating 18 RCTs with 1308 HCC patients, combining TACE with ginsenosides, bioactive compound of Radix Ginseng, could significantly improve objective response rate (RR = 1.39, 95% CI: 1.20 – 1.61), disease control rate (RR = 1.21, 95% CI: 1.12 – 1.30), and quality of life (QoL) (RR = 1.54, 95% CI: 1.25 – 1.90). Interestingly, ginsenosides also alleviated adverse effects of TACE, as evidenced by lower risks of hyperbilirubinemia, fatigue, nausea, pyrexia, ache, anorexia, leukopenia, thrombocytopenia, and myelosuppression [49]. One pitfall of this meta-analysis is that most of the incorporated RCTs are single-centered trials, which have intrinsic single-center bias. Some of the RCTs are of small sample size, which may potentially undermine the scientific significance. Besides Radix Ginseng, we have summarized other TCM that were used in combination with TACE, including Jinlong Capsule, Compound Kushen Injection, Huachansu Tablet, Kanglaite Injection, Jiedu Granule, and Fuzheng Jiedu Formula [45, 50–54].

Notably, some TCM are viewed as important early intervention approaches for the prevention of liver cancer [55]. HCC tend to develop in chronic liver disease, which commonly resulted from chronic hepatitis B or C virus infections. Oxymatrine, a natural compound derived from Chinese herb Radix Sophorae, displayed antiviral activity in 216 chronic hepatitis B patients [56]. Another natural compound Silibinin also demonstrated potent antiviral effects against chronic hepatitis C [57]. According to a RCT incorporating 1000 patients with chronic hepatitis B, TCM formula Biejia Ruangan Compound could effectively lower the incidence of HCC (1-, 3-, 5-, 7-year cumulative incidence of HCC 0.2%, 1.0%, 1.9%, 4.7% *vs.* 0.4%, 2.4%, 4.6%, 9.3%, HR = 0.489, 95% CI: 0.288 – 0.832, *p* = 0.008) and liver related mortality (0, 0.2%, 0.2%, 0.2% *vs.* 0, 0.5%, 1.0%, 2.2%, HR = 0.101, 95% CI: 0.013 – 0.797, *p* = 0.030) in combination with an antiviral drug entecavir [58]. This study provides evidence that the integrative approach of combining TCM with conventional antiviral therapy may offer a promising strategy for improving outcomes in patients with chronic hepatitis B, potentially reducing the burden of HCC and associated mortality. The RCT possesses a multicenter, large-scale, double-blind and placebo-controlled design, establishing robust evidence that Biejia Ruangan Compound can mitigate the risk of HCC in patients with chronic hepatitis B.

### Biliary cancer

Biliary tract cancer (BTC) is the second most common type of hepatobiliary malignancy, including gallbladder cancer (GBC), intrahepatic cholangiocarcinoma (ICC), and extrahepatic cholangiocarcinoma (ECC). BTC is challenging to detect in its early stages and only about 20% of patients are diagnosed during the resectable period [59]. Additionally, BTC demonstrates a high degree of invasiveness and insensitivity to conventional treatments, resulting in a poor prognosis [2, 60, 61]. In the advanced unresectable BTC, chemotherapy is considered the cornerstone of treatment [62]. For over a decade, the combination of gemcitabine and cisplatin has been considered the established standard of care for first-line systemic therapy in the treatment of BTC [63]. The recent TOPAZ-1 trial has shown that the addition of durvalumab to the standard regimen significantly improves OS (median: 12.8 *vs*. 11.5 months, HR = 0.80, *p* = 0.021, 2-year OS rate: 24.9% *vs*. 10.4%) and it has been approved as a first-line treatment [64]. Despite advances in first-line treatments for BTC, there are still limitations such as poor response rates and tolerability issues, indicating optimized treatment is needed.

TCM has shown promise as an adjunctive therapy in the treatment of BTC, with clinical evidence supporting its efficacy and safety [65, 66]. Huachansu, a TCM derived from the dried venom of *Bufo bufo gargarizans* Cantor or *Bufo melanostictus* Schneider, has been widely utilized in the treatment of cancer. Co-administration of Huachansu with chemotherapy or radiotherapy has been found to exert a synergistic effect, potentiating the therapeutic efficacy of conventional interventions while concurrently ameliorating their toxicity profile [67]. Clinical research data has shown that the combination of gemcitabine-oxaliplatin with Huachansu compared to chemotherapy alone improved progression-free survival (PFS) to 5.8 month (95% CI: 4.5 – 7.1 months) and an OS of 10.5 months [68]. Additionally, the combination of Huachansu Capsule with the S-1 and oxaliplatin (SOX) regimen in the treatment of advanced GBC demonstrated a significantly higher overall response rate compared to the control group (80.6% *vs*. 54.8%, *p* < 0.05), with low incidence of adverse reactions [69]. The combination of Huachansu Capsule with the SOX regimen has been proven to be effective in the treatment of advanced gallbladder cancer, with low incidence of adverse reactions, and deserves further in-depth research.

In addition, Shugan Lidan Decoction, composed of a combination of various Chinese herbal ingredients, has consistently shown significant therapeutic effects in the treatment of chronic cholecystitis and gallstones [70]. Recent clinical data has demonstrated its potential as an adjunctive treatment for gallbladder cancer. The combination of Shugan Lidan Decoction with stereotactic body radiotherapy (SCRT) in the treatment of advanced bile duct cancer significantly improves the 6-month and 1-year OS rates and median survival time compared to SCRT alone (90.32%, 58.06%, and 13.69 months *vs*. 67.74%, 35.48%, and 9.16 months, *p* < 0.05) [71]. This indicates that Shugan Lidan Decoction effectively prolong the survival of patients and has the potential to be used as an adjuvant therapy.

Kanglaite Injection is a broad-spectrum anti-tumor TCM preparation that has received approval for cancer treatment by the NMPA [72, 73]. In a RCT comparing Kanglaite Injection with cisplatin, doxorubicin, and 5-fluorouracil treatment in patients with cholangiocarcinoma, it was found that the combination therapy improved the effective rate (80.00% *vs*. 28.00%, *p* < 0.05), Karnofsky performance status (KPS) score (*p* < 0.05), and immunity (*p* < 0.05) [74]. Cidan Capsule works as an adjunctive chemotherapy agent by removing blood stasis, relieving toxins, and nourishing blood [75, 76]. In a RCT of elderly advanced gallbladder cancer, patients treated with a combination of Cidan Capsule, oxaliplatin, and tegafur significantly improved the effective rate (85.4% *vs*. 70.8%, *p* < 0.05) and KPS score (82.6 ± 10.9 *vs*. 75.2 ± 9.6, *p* < 0.05) [77]. Fuzheng Kangai Formula is a formulation composed of 12 TCMs and has been consistently used as an adjunctive treatment strategy for lung cancer patients [78]. Recent clinical studies have shown promising efficacy in gallbladder cancer as well. When used in combination with the gemcitabine and cisplatin regimen, Fuzheng Kangai Formula has been found to improve the effective rate (83.3% *vs*. 60.0%, *p* = 0.045) and PFS (9.2, 95% CI: 8.02 – 10.489 *vs*. 7.6, 95% CI: 6.698 – 8.525 months, *p* = 0.030) [79]. Nonetheless, the aforementioned clinical trials are constrained by single-center small participant cohorts and the absence of placebo controls, highlighting the imperative for further placebo controlled double-blind trials to corroborate the clinical efficacy of TCM in treating BTC.

### Pancreatic cancer

Pancreatic cancer (PCC), among which pancreatic adenocarcinoma represents the most frequent type, has been recognized as a malignant tumor with high morbidity and mortality rates (top 10 worldwide and top 3 in America) [80, 81]. PCC usually displays little signs and symptoms in the early stage but rapid progression to the advanced stage, which lead to difficulty in early diagnosis and poor prognosis [82, 83]. Conventional therapy for PCC, including surgery, chemotherapy, and radiotherapy, does not always exhibit satisfied clinical outcome, sometimes offering only marginal remission and survival rate [84, 85]. The five-year survival rate of PCC remains at approximately 10% or even lower than 5% [86, 87]. Drug resistance and adverse reaction of the current first-line therapy, such as gemcitabine, has also attracted wide attention [88, 89]. In this context, it is worth exploring more effective therapy for PCC treatment.

TCM as complementary or alternative medicine has been commonly applied for PCC treatment in China and other Asian countries and has shown favorable clinical efficacy and safety as revealed by RCTs as well as systematic review and meta-analysis [90–93]. Of note, in a meta-analysis of 29 RCTs involving 1808 patients with unresectable advanced PCC, TCM prescriptions combined with conventional therapy compared to conventional therapy improved 6-month survival rate (RR = 1.58, 95% CI: 1.05 – 2.37, *p* = 0.03), 1-year survival rate (RR = 1.85, 95% CI: 1.49 – 2.31, *p* < 0.00001), objective response rate (RR = 1.42, 95% CI: 1.26 – 1.59, *p* < 0.00001), disease control rate (RR = 1.25, 95% CI: 1.12 – 1.39, *p* < 0.0001), clinical benefit rate (RR = 1.55, 95% CI: 1.30 – 1.84, *p* < 0.00001), and quality of life (categorical data: RR = 1.25, 95% CI: 1.12 – 1.39, *p* = 0.0002; continuous data: MD = 4.36, 95% CI: -2.57 – 11.28, *p* = 0.22), and decreased the incidence of gastrointestinal reaction (RR = 0.36, 95% CI: 0.21 – 0.63, *p* = 0.0003) and grade III-IV leukopenia (RR = 0.71, 95% CI, 0.57 – 0.90, *p* = 0.004).

In another meta-analysis of 31 RCTs involving 1,989 patients with advanced PCC, TCM prescriptions combined with chemotherapy compared to chemotherapy alone improved objective response rate (RR = 1.64, 95% CI: 1.43 – 1.88, *p* < 0.00001), disease control rate (RR = 1.29, 95% CI: 1.21 – 1.38, *p* < 0.00001), and quality of life (continuous data: SMD = 0.81, 95% CI: 0.44 – 1.18, *p* < 0.0001; dichotomous data: RR = 1.44, 95% CI: 1.22 – 1.70, *p* < 0.0001), decreased the level of carbohydrate antigen 19–9 (SMD = -0.46, 95%: CI -0.90 – -0.02, *p* = 0.04) and carcinoembryonic antigen (SMD = -0.55, 95% CI: -0.93 – -0.17, *p* = 0.004), and reduced the risk of leukopenia (RR = 0.43, 95% CI: 0.27 – 0.70, *p* = 0.0005), thrombopenia (RR = 0.54, 95% CI: 0.35 – 0.84, *p* = 0.006), hemoglobinopenia (RR = 0.61, 95% CI: 0.40 – 0.94, *p* = 0.02), and gastrointestinal reaction (RR = 0.33, 95% CI: 0.12 – 0.90, *p* = 0.03) [94].

In detail, these TCM prescriptions are mainly injections, including Aidi Injection, Astragalus Polysaccharide Injection, Compound Kushen Injection, Disodium Cantharidinate and Vitamin B6 Injection, Huanchansu Injection, Javanica Oil Emulsion Injection, Kangai Injection, Kanglaite Injection, Shenmai Injection, Shenqi Fuzheng Injection, etc. Among these injections, Aidi Injection, Compound Kushen Injection, Kangai Injection, and Kanglaite Injection, has been documented with solid evidence. As revealed in two network meta-analysis, Aidi Injection plus chemotherapy vs. chemotherapy could reduce leukopenia [95]; improve clinical efficacy and reduce thrombocytopenia [96]. Three network meta-analysis reported that Compound Kushen Injection plus chemotherapy/radiotherapy vs. control could improve performance status [95]; improve performance status, increase pain relief rate [96]; improve overall response rate and KPS score, reduce leukopenia and nausea/vomiting [97]. Three network meta-analysis demonstrated that Kangai Injection plus chemotherapy vs. control could improve performance status [95]; improve clinical efficacy and performance status [96]; improve clinical benefit rate [98]. Six network meta-analysis supported that Kanglaite Injection plus chemotherapy/radiotherapy vs. control could improve performance status [95]; improve clinical efficacy and performance status, reduce leukopenia, thrombocytopenia, and gastrointestinal reactions, increase pain relief rate [96]; increase KPS score [97]; reduce leukopenia [98]; improve 1-year overall survival, overall response, disease control rate, life quality improvement rate, pain relief rate, and weight gain rate [99]; improve effective rate, life quality improvement rate, pain relief rate, and weight gain rate, reduce incidence of bone marrow depression, liver dysfunction, and kidney dysfunction [100]. Based on these findings, Kanglaite Injection may be one of the optimum choices as adjuvant therapy for PCC treatment. However, some of the discussed meta-analysis incorporated small cohort RCTs, which may have larger effect sizes, leading to an overestimation of the treatment effect in the meta-analysis.

For further information and quick reference, the clinical efficacy and safety as well as the medicinal constituent of TCM for the treatment of hepatic, biliary, and pancreatic cancer have been displayed in Tables 1 and 2, respectively. In addition, the original resource of the medicinal constituent of TCM has also been presented in Supplementary Information (SI)**-**Table 1.

**Table 1:** Clinical efficacy and safety of TCM for the treatment of hepatic, biliary, and pancreatic cancer

TCM	Comparison	Participant	Design	Outcome	Ref
Biejia Ruangan Compound	Biejia Ruangan Compound + entecavir *vs.* placebo + entecavir	1000 patients with chronic hepatitis B	RCT	Decrease 1-, 3-, 5-, 7-year cumulative incidence of HCC and liver-related deaths	[58]
Compound Kushen Injection	Compound Kushen Injection + TACE *vs*. TACE	1388 patients (18 RCTs) with HCC	Meta	Improve tumor response, 1-year OSR, 2-year OSR, KPS, and Child–Pugh	[51]
Fuzheng Jiedu Xiaoji Formula	Fuzheng Jiedu Xiaoji Formula + TACE *vs*. TACE	291 patients with HBV-related HCC	RCT	Prolong OS in BCLC A or B stage and PFS in BCLC B stage	[54]
Ginsenosides	Ginsenosides + TACE *vs*. TACE	1308 patients (18 RCTs) with HCC	Meta	Improve ORR, DCR, QoL, 1-year OS, and 2-year OS	[49]
Ginsenoside Rg3	Ginsenoside Rg3 + TACE *vs*. TACE	228 patients with advanced HCC	RCT	Improve OS, 6-month OSR, 12-month OSR, and DCR	[101]
Huachansu Tablet	Huachansu Tablet + TACE *vs*. TACE	112 patients with unresectable HCC	RCT	Prolong median PFS and OS	[102]
Huaier Granule	Huaier Granule *vs*. no further treatment	1044 patients with HCC	Multi-center RCT	Improve RFS, RFS rate, OSR, and ERR	[40]
Huaier Granule	Huaier Granule *vs*. non-Huaier Granule	826 patients with HCC	Cohort study	Decrease mortality risk, increase 3-year OSR	[39]
Icaritin	Icaritin single arm	20 patients with HCC	Phase I, single arm	Achieve clinical benefit rate of 46.7%, median TTP of 141 days, median OS of 192 days, without drug-related adverse events over Grade 3	[103]
Jianpi Huayu Therapy	Jianpi Huayu Therapy + hepatectomy *vs*. hepatectomy	120 patients with HCC	RCT	Improve 1-, 3-, and 5-year DFS rate, medium DFS, OSR, and median OS	[104]
Jiedu Granule	Jiedu Granule + TACE + GKR *vs*. TACE + GKR	376 patients with HCC complicated with PVTT	RCT	Prolong median OS	[53]
Jinlong Capsule	Jinlong Capsule + TACE *vs*. TACE	1725 patients (19 RCTs) with HCC	Meta	Prolong 6-month, 24-month, and 36-month OS, improve ORR and DCR	[50]
Jinlong Capsule	Jinlong Capsule + conventional treatment *vs*. conventional treatment	2488 patients (29 RCTs) with HCC	Meta	Prolong 6-month, 12-month, 24-month, and 36-month OS, improve ORR and DCR	[105]
Kangai Injection	Kangai Injection + conventional treatment *vs*. conventional treatment	2501 patients (35 RCTs) with HCC	Meta	Improve ORR, DCR, and QoL	[106]
Kanglaite Injection	Kanglaite Injection + TACE *vs*. TACE	608 patients (9 RCTs) with HCC	Meta	Improve ORR and QoL, relief pain	[52]
PHY906	PHY906 + capecitabine single arm	39 patients with HCC	Phase II, single arm	Median PFS was 1.5 months, and median OS was 6.0 months, with a 6‐month survival rate of 51.3%	[107]
PHY906	PHY906 + capecitabine single arm	42 patients with HCC	Phase I/II, single arm	More than 60% of patients benefit, with a median OS of 9.2 months	[108]
Cidan Capsule	Cidan Capsule + mFOLFOX6 *vs*. mFOLFOX6	82 patients with cholangiocarcinoma	RCT	Improve total survival time, ORR, 1-year OSR, 2-year OSR, and 3-year OSR	[109]
Cidan Capsule	Cidan Capsule + SOX *vs*. SOX	96 patients with advanced gallbladder cancer	RCT	Improve effective rate and KPS score	[77]
Fuzheng Kangai Formula	Fuzheng Kangai Formula + GPR *vs*. GPR	60 patients with gallbladder cancer	RCT	Improve effective rate and PFS	[79]
Huachansu Capsule	Huachansu Capsule + SOX *vs*. SOX	62 patients with advanced gallbladder cancer	RCT	Improve total effective rate	[69]
Kanglaite Injection	Kanglaite Injection + PAF *vs*. PAF	55 patients with cholangiocarcinoma	RCT	Improve effective rate, KPS, and immunity	[74]
Shugan Lidan Decoction	Shugan Lidan Decoction + SCRT *vs*. SCRT	62 patients with advanced cholangiocarcinoma	RCT	Improve effective rate, median OS, 6-month OSR, and 1-year OSR	[71]
Aidi Injection	Aidi Injection + Chemo vs. Chemo	1329 patients (22 RCTs) with PCC	NMA	Reduce leukopenia	[95]
Aidi Injection	Aidi Injection + Chemo vs. Chemo	2011 patients (33 RCTs) with PCC	NMA	Improve clinical efficacy, reduce thrombocytopenia	[96]
Compound Kushen Injection	Compound Kushen Injection + Chemo vs. Chemo	2011 patients (33 RCTs) with PCC	NMA	Improve performance status, increase pain relief rate	[96]
Compound Kushen Injection	Compound Kushen Injection + Radio vs. Radio	1199 patients (18 RCTs) with advanced PCC	NMA	Improve ORR and KPS score, reduce leukopenia and nausea/vomiting	[97]
Kangai Injection	Kangai Injection + Chemo vs. Chemo	2011 patients (33 RCTs) with PCC	NMA	Improve clinical efficacy and performance status	[96]
Kangai Injection	Kangai Injection + gemcitabine vs. gemcitabine	808 patients (14 RCTs) with PCC	NMA	Improve clinical benefit rate	[98]
Kanglaite Injection	Kanglaite Injection + Chemo vs. Chemo	2011 patients (33 RCTs) with PCC	NMA	Improve clinical efficacy and KPS, reduce gastrointestinal reactions, leukopenia, and thrombocytopenia	[96]
Kanglaite Injection	Kanglaite Injection + Radio-Chemo vs. Radio-Chemo	960 patients (16 RCTs) with advanced PCC	Meta	Improve 1-year OS, DCR, overall response, QoL improvement rate, pain relief rate, and weight gain rate	[99]
Kanglaite Injection	Kanglaite Injection + Chemo vs. Chemo	531 patients (10 RCTs) with advanced PCC	Meta	Improve effective rate, QoL improvement rate, pain relief rate, and weight gain rate	[100]
Chaihu Liujunzi Decoction	Chaihu Liujunzi Decoction + S-1 vs. S-1	137 patients with advanced PCC	RCT	Improve DCR	[110]
Gexia Zhuyu Decoction	Gexia Zhuyu Decoction + gemcitabine vs. gemcitabine	102 patients with PCC	RCT	Improve effective rate	[111]
Qingyi Huaji Decoction	Qingyi Huaji Decoction + IAC vs. IAC	30 patients with middle-advanced PCC	RCT	Improve effective rate, 6-month OSR, 12-month OSR, and QoL score	[112]
Xiaochaihu Decoction	Xiaochaihu Decoction + radio vs. radio	32 patients with middle-advanced PCC	RCT	Improve effective rate, PFS, OS, 1-year OSR	[113]
Yinchenhao Decoction	Yinchenhao Decoction + S-1 vs. S-1	60 patients with advanced PCC	RCT	Improve DCR and KPS score	[114]

*aHR* adjusted hazard ratio, *BCLC* barcelona Clinic Liver Cancer, *Chemo* chemotherapy, *Chemo-Radio* chemoradiotherapy, *CI* confidential interval, *CrI* credible interval, *DCR* disease control rate, *DFS* disease free survival, *ERR* extrahepatic recurrence rate, *GKR* gamma knife radiosurgery, *GPR*, gemcitabine and cisplatin regimen, *HBV* hepatitis B virus, *HCC* hepatocellular carcinoma, *HR* hazard ratio, *IAC*, intra-arterial chemotherapy, *KPS* karnofsky performance status, *Meta* meta-analysis, mFOLFOX6, 5-fluorouracil, leucovorin, and oxaliplatin, *NMA* network meta-analysis, *OR* odds ratio, *ORR* overall response rate, *OS* overall survival, *OSR* overall survival rate, *PAF* cisplatin, doxorubicin, and 5-fluorouracil, *PCC* pancreatic cancer, *PFS* progression free survival, *PVTT* portal vein tumor thrombosis, *Radio* radiotherapy, *RCT* randomized controlled trial, *RFS* recurrence-free survival, *RR* relative risk; S-1 (TGO), tegafur, gimeracil, and oteracil; *SCRT* stereotactic conformal radiotherapy, *SOX* S-1 plus oxaliplatin, *TACE* transcatheter arterial chemoembolization, *TCM* traditional Chinese medicine, *TTP* time-to-progression

**Table 2:** Medicinal constituent of TCM for the treatment of hepatic, biliary, and pancreatic cancer

TCM	Medicinal constituent
Aidi Injection	Ban-Mao 60 g, Ren-Shen 600 g, Huang-Qi 1200 g, Ci-Wu-Jia 100 g
Biejia Ruangan Compound	Bie-Jia 120 g, Chi-Shao 85 g, Dang-Gui 50 g, San-Qi 50 g, Dang-Shen 85 g, Huang-Qi 85 g, Yang-Tai-Pan 22.5 g, Dong-Chong-Xia-Cao 25 g, Ban-Lan-Gen 140 g, Lian-Qiao 140 g, E-Zhu 25 g
Chaishao Liujunzi Decoction	Chai-Hu 15 g, Bai-Shao 15 g, Dang-Shen 20 g, Bai-Zhu 15 g, Fu-Ling 15 g, Gan-Cao 5 g, Chen-Pi 10 g, Fa-Ban-Xia 10 g
Cidan Capsule	Shan-Ci-Gu 50 g, E-Zhu 160 g, Ma-Qian-Zi 13 g, Feng-Fang 50 g, Ya-Dan-Zi 50 g, Ren-Gong-Niu-Huang 12 g, Jiang-Can 80 g, Dan-Shen 80 g, Huang-Qi 160 g, Dang-Gui 80 g, Bing-Pian 3 g
Compound Kushen Injection	Ku-Shen 140 g, Bai-Tu-Fu-Ling 60 g
Fuzheng Jiedu Xiaoji Formula	Dang-Shen 15 g, Huang-Qi 15 g, Bai-Zhu 15 g, Fu-Ling 15 g, Nan-Sha-Shen 15 g, Mai-Dong 15 g, Dang-Gui 15 g, Shu-Di-Huang 15 g, Qi-Ye-Yi-Zhi-Hua 15 g, E-Zhu 15 g, Ban-Xia 9 g
Fuzheng Kangai Formula	Huang-Qi 15 g, Nv-Zhen-Zi 15 g, Ling-Zhi 15 g, Teng-Li-Gen 15 g, Dan-Shen 15 g, E-Zhu 15 g, Mu-Li (Xian) 15 g, Shui-Zhi 6 g
Gexia Zhuyu Decoction	Huang-Qi 20 g, Tao-Ren 10 g, Chi-Shao 10 g, Fu-Ling 10 g, Xiang-Fu 10 g, Mu-Dan-Pi 10 g, Wu-Yao 10 g, Dang-Gui 10 g, Bai-Zhu 10 g, Hong-Hua 6 g, Chuan-Xiong 6 g, Wu-Ling-Zhi 6 g, Gan-Cao 6 g, Zhi-Qiao 6 g
Huachansu Capsule/Tablet	Chan-Su
Huaier Granule	Huai-Er
Jianpi Huayu Therapy	Ren-Shen 20 g, Bai-Zhu 15 g, Fu-Ling 15 g, Gan-Cao 6 g, Chai-Hu 15 g, Shan-Yao 12 g, Mu-Dan-Pi 10 g, Dan-Shen 15 g, Jiang-Huang 10 g, E-Zhu 10 g
Jiedu Granule	Zi-Shen 28.1 g, Mao-Ren-Shen 28.1 g, Ji-Nei-Jin 11.2 g, Shan-Ci-Gu 11.2 g
Jinlong Capsule	Shou-Gong (Xian) 1500 g, Jin-Qian-Bai-Hua-She (Xian) 750 g, Qi-She (Xian) 750 g
Kangai Injection	Ren-Shen 1 g, Huang-Qi 3 g, oxymatrine 100 mg
Kanglaite Injection	Yi-Yi-Ren
Ginsenosides/Ginsenoside Rg3	Ren-Shen
PHY906	Huang-Qin 9 g, Gan-Cao 6 g, Bai-Shao 6 g, Da-Zao 6 g
Qingyi Huaji Decoction	Ling-Zhi 30 g, Dou-Kou 5 g, Ban-Zhi-Lian 30 g, Yi-Yi-Ren (Xian) 30 g, She-Liu-Gu 15 g, Jiao-Gu-Lan 30 g, Bai-Hua-She-She-Cao 15 g
Shenqi Fuzheng Injection	Dang-Shen 40 g, Huang-Qi 40 g
Shugan Lidan Decoction	Chai-Hu 15 g, Bai-Shao 15 g, Chuan-Xiong 10 g, Yu-Jin 10 g, Huang-Qin 10 g, Qing-Ban-Xia 10 g, Jin-Qian-Cao 15 g, Zhi-Zi 15 g, Yin-Chen 30 g, Long-Dan 15 g, Ling-Xiao-Hua 15 g, Shui-Hong-Hua-Zi 10 g, Teng-Li-Gen 15 g, Long-Kui 10 g, Gan-Cao 10 g
Xiaochaihu Decoction	Chai-Hu 15 g, Bai-Hua-She-She-Cao 15 g, Shan-Ci-Gu 15 g, Teng-Li-Gen 15 g, Yan-Hu-Suo 9 g, Huang-Qin 9 g, Ren-Shen 9 g, Ban-Xia 9 g, Sheng-Jiang 9 g, Fo-Shou 6 g, Gan-Cao 6 g, Da-Zao 12 pieces
Yinchenhao Decoction	Yin-Chen 25 g, Zhi-Zi 10 g, Da-Huang 5 g, Dang-Shen 20 g, Huang-Qi (Xian) 20 g, Bai-Zhu (Xian) 30 g, Fu-Ling 20 g, Teng-Li-Gen 15 g, She-Liu-Gu 15 g, Tu-Fu-Ling 15 g, Ji-Nei-Jin 30 g, Chui-Pen-Cao 30 g
Icaritin	Yin-Yang-Huo /Wu-Shan-Yin-Yang-Huo

## Representative formulae, bioactive compounds, and therapeutic targets

**Fig. 1:**
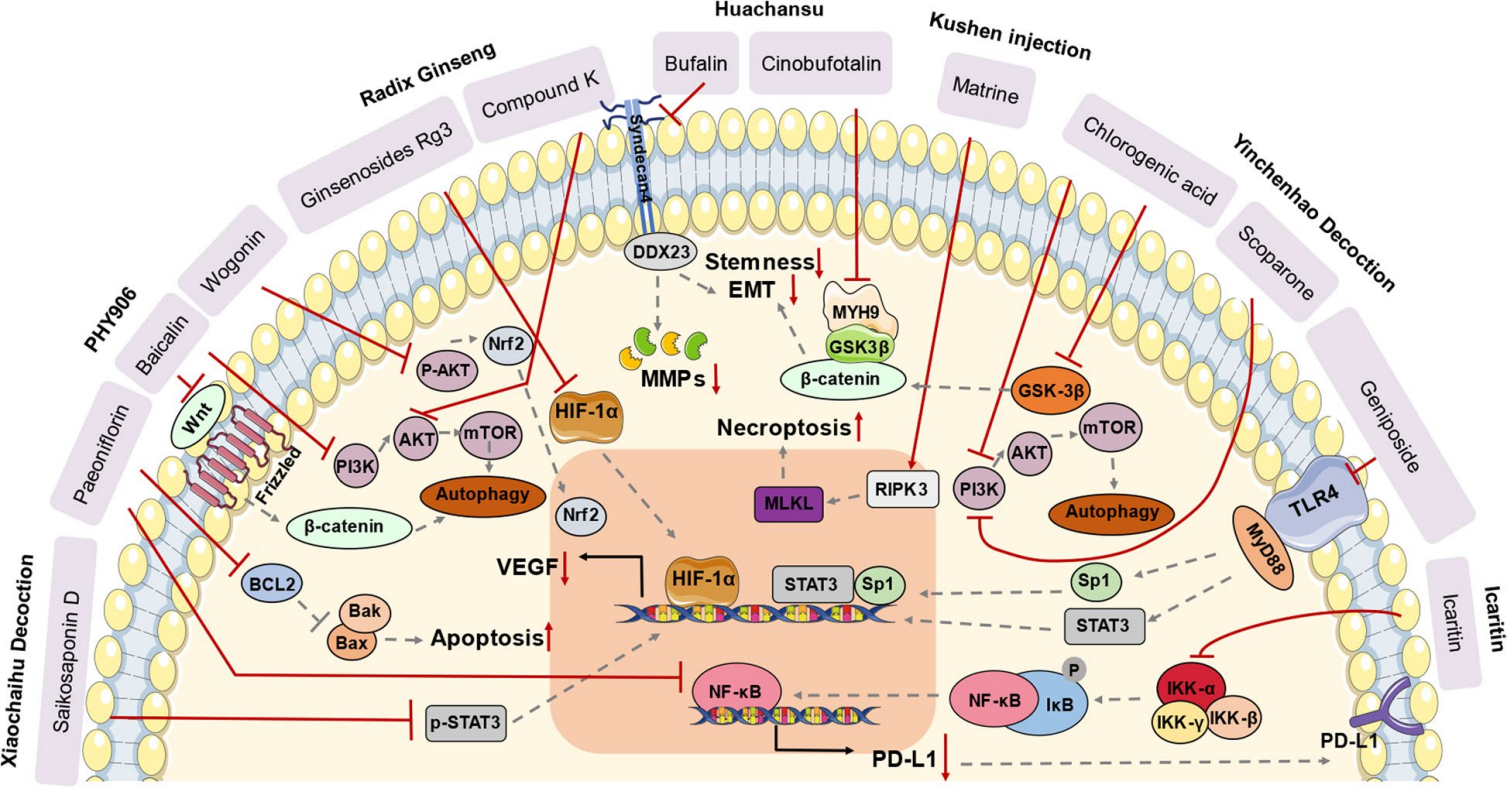
The bioactive compounds identified from several representative TCM formula with their potential target and pharmacological action. EMT, epithelial-to-mesenchymal transition; MMPs, matrix metalloproteinases; VEGF, vascular endothelial growth factor

In this section, we aim to systematically reviewed the single bioactive compounds identified from the TCM that have garnered substantial evidence for their efficacy in treating hepatic, biliary, and pancreatic cancer. The pharmacological mechanism and target were also comprehensively summarized (Fig. 1).

### Huaier granule

**Fig. 2:**
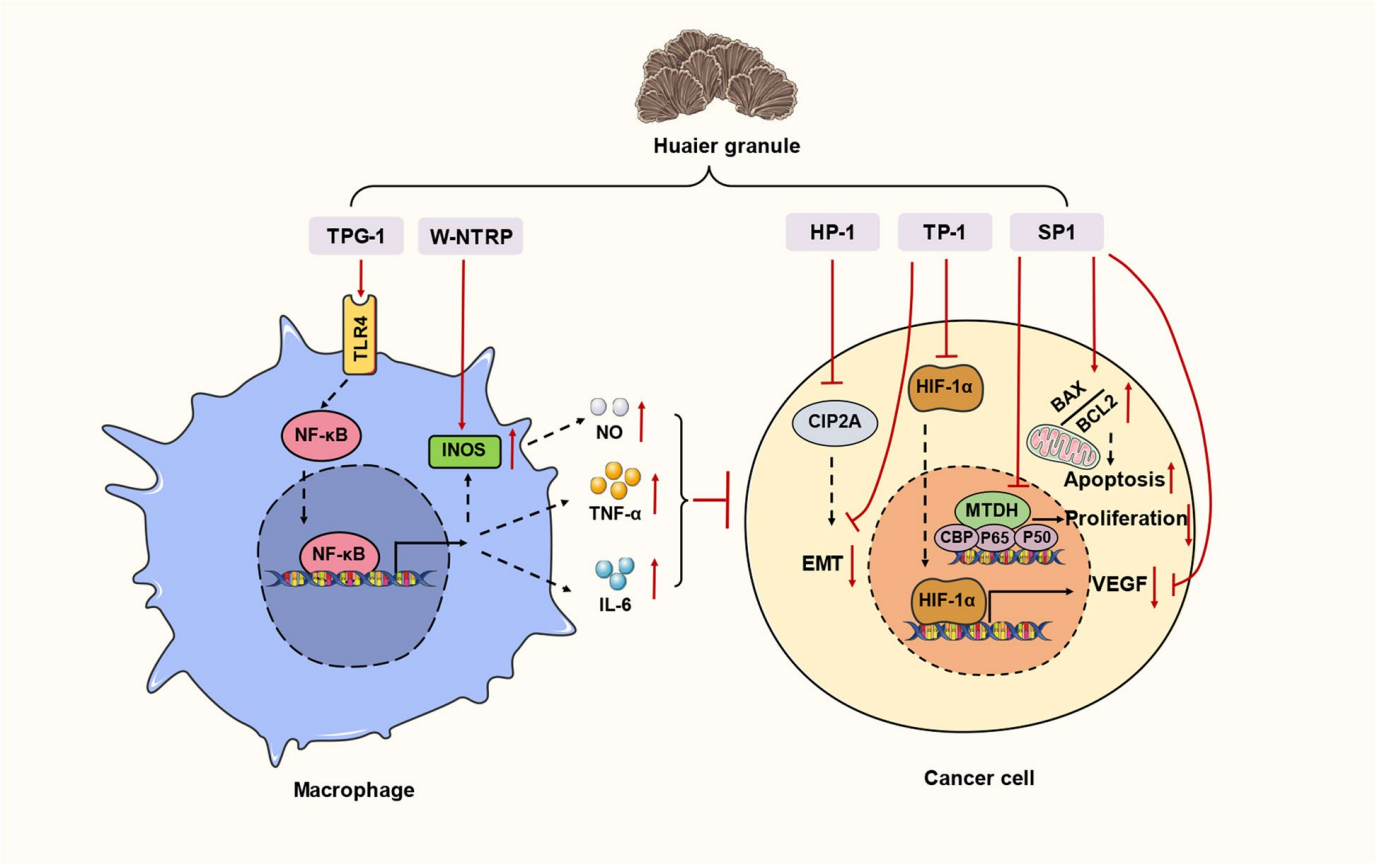
Pharmacological mechanism of bioactive polysaccharides identified from Huaier granule

Huaier Granule is made from the aqueous extract of Poria Robiniophila (*Poria robiniophila* (Murrill) Ginns), which is a sandy beige officinal fungus grown on trunks of hard wood trees. As an ancient Chinese medicine, Huaier has been widely used in China for 1600 years [115]. By virtue of its potent broad-spectrum antitumor effects, Huaier granule has been approved by NMPA to be used alone or combined with other drugs in treatment of multiple types of cancer [116]. The bioactive component of Huaier aqueous extract is proteoglycan, which contains 41.5% of polysaccharides, 12.93% of amino acids and 8.72% of water [117]. The remarkable clinical efficacy of Huaier Granule prompted extensive research aimed at investigating the precise bioactive compound responsible for its therapeutic effects. To date, 5 homogenous bioactive polysaccharides or proteoglycans have been purified from Huaier, including TPG-1, HP-1, W-NTRP, TP-1, and SP1. These compounds with tumoricidal cytotoxicity and immunoregulatory properties collectively contribute to the anti-tumor pharmacological action of Huaier Granule (Fig. 2).

#### TPG-1

TPG-1 is a proteoglycan with a molecular mass of 55.9 kDa composed by 43.9% of carbohydrates and 41.2% of protein. TPG-1 inhibited the growth of xenograft hepatoma in nude mice and exhibited intrinsic immunoregulatory activity. Specifically, TPG-1 potentiated the production of inflammatory cytokines, including nitric oxide (NO), tumor necrosis factor α (TNF-α), and interleukin-6 (IL-6), in macrophages by activating toll-like receptor 4/nuclear factor kappa B/mitogen-activated protein kinase (TLR4/NF-κB/MAPK) signaling in macrophages [118].

#### HP-1/TP-1

HP-1 and TP-1 are two homogenous polysaccharides purified by similar chromatographic method. HP-1 is around 30 kDa and TP-1 is around 2300 kDa. HP-1 could inhibit the epithelial-mesenchymal transition (EMT) process during tumor invasion by targeting cellular inhibitor of PP2A (CIP2A) and its anti-oxidative activity enable protection against cisplatin induced kidney damage [119, 120]. TP-1 demonstrated the ability to effectively impede the progression of HCC by inhibiting hypoxia inducible factor 1 subunit alpha/vascular endothelial growth factor (HIF-1α/VEGF) mediated angiogenesis and boosting immunity [121, 122].

#### W-NTRP

W-NTRP is an arabinogalactan mainly composed by galactose and arabinose, with a molecular weight of 25 kDa. W-NTRP demonstrated cytotoxicity against human cholangiocarcinoma cell lines while exhibiting no cytotoxic effects on normal cells. W-NTRP also stimulated immunity by increasing inducible NO synthase (iNOS) activity in macrophages [123].

#### SP1

SP1 is another homogenous polysaccharide purified from Huaier with a molecular weight of 56 kDa. SP1 suppressed the proliferation of both liver cancer cells and breast cancer cells. This inhibitory effect can be attributed, at least in part, to the downregulation of the oncogenic metadherin (MTDH) protein expression [124, 125].

### PHY906

As a standardized pharmaceutical grade agent, PHY906 is a spray-dried aqueous extract derived from a TCM formulation Huangqin Decoction, which has been used for over 1800 years for treating gastrointestinal diseases such as including diarrhea, abdominal cramps, and vomiting [126]. PHY906 is composed by 4 herbs, namely Radix Scutellariae, Radix Glycyrrhizae, Radix Paeoniae Rubra, and Fructus Jujubae. Preclinical evidence supported the notion that PHY906 possesses the ability to mitigate chemotherapy-induced gastrointestinal toxicity [127], while concurrently augmenting the therapeutic efficacy of several commonly used anticancer agents. These agents include irinotecan, 5-fluorouracil, capecitabine, sorafenib, irinotecan, and immune checkpoint inhibitors, as demonstrated in a diverse range of tumor-implanted animal models and clinical trials [128–133]. The antitumor activity of PHY906 stems from a multi-faceted mechanism, involving the inhibition of NF-κB, matrix metalloproteases (MMPs), multi-drug resistant protein (MDR), and cytochrome P450 (CYP450), as well as the modulation of numerous cytokines. Thus far, a total of 64 bioactive compounds have been identified from PHY906, including flavonoids, triterpene saponins, and monoterpene glycosides. Notably, several of these compounds have demonstrated significant pharmacological effects against tumors, highlighting their potential as promising candidates for further development [134–136].

#### Flavonoids and flavonoid glycosides

Flavonoids and flavonoid glycosides are the major active components of Radix Scutellariae as well as PHY906. Ye et al. identified six free flavones from PHY906, of which baicalein, wogonin, and oroxylin A, along with their corresponding glycosylated forms baicalin, wogonoside, and oroxylin-A-glucoside, are representative compounds with antitumor bioactivity [134]. Both baicalin and its aglycone baicalein demonstrated anti-inflammatory, antioxidant, and antitumor effects [137]. Baicalin restrains cancer cell proliferation by targeting apoptotic pathway and tumor growth related pathway such as phosphoinositide 3-kinase/protein kinase B/mammalian target of rapamycin (PI3K/Akt/mTOR) and Wnt/β-catenin signaling [138–140]. Recent study illustrated that baicalin could induce ferroptosis in cancer cells and reverse resistance towards anti-PD-1 treatment [141, 142]. Similarly, baicalein has been shown to trigger cancer cell death by arresting cell cycle and inducing DNA damages [143, 144]. Wogonin was reported to be widely used to boost chemotherapy efficacy through exacerbating DNA damages and inhibiting Akt and Nrf2 (nuclear factor erythroid 2-related factor 2) signaling [145–149]. Its glycosylated form, wogonoside, also demonstrated potent cytotoxicity against both solid tumor and blood cancer [150, 151]. Oroxylin A possesses broad antitumor effects across different cancer [152]. In HCC, oroxylin A was identified as a novel inhibitor of cyclin-dependent kinase 9 (CDK9) and transketolase [153–155]. By targeting these molecular targets, oroxylin A effectively inhibits the progression of HCC in preclinical mice models.

#### Glycyrrhizic acid

Triterpene saponins are characteristic constituents of Radix Glycyrrhizae. Among the 20 identified triterpenoids, glycyrrhizic acid serves as the principal compound and has been included in the quality control standard established for Radix Glycyrrhizae by the Chinese Pharmacopoeia [156, 157]. Glycyrrhizic acid is a specific inhibitor of high mobility group box 1 (HMGB1), which regulates DNA structure but can be released into the extracellular space, playing a tumor-promoting role in HCC by stimulating tumorigenesis, proliferation, and metastasis [158–161]. Extracellular HMGB1 inhibition by glycyrrhizic acid efficiently boosted anti-PD-1 efficacy by remodeling the tumor microenvironment [162].

#### Paeoniflorin

Monoterpene glycosides are the major bioactive constituents of Radix Paeoniae Rubra. The main monoterpene glycosides include paeoniflorin, albiflorin, oxypaeoniflorin, benzoylpaeoniflorin, and hydroxybenzoylpaeoniflorin, among which paeoniflorin is the principal bioactive compound contributing to the medicinal properties of Radix Paeoniae Rubra [163]. Paeoniflorin exhibits an antitumor effect by inhibiting NF-κB and B-cell lymphoma 2 (Bcl-2) signaling, leading to the activation of cell apoptosis-associated pathways [164]. Interestingly, paeoniflorin was corroborated to downregulating programmed death-ligand 1 (PD-L1) expression in HCC, thereby suppressing the immune evasion ability of HCC and impeding its progression [165, 166].

### *Radix* Ginseng

The root of the *Panax ginseng* C.A.Mey., commonly known as Radix Ginseng, has been traditionally employed as a medicinal herb in Asian countries, primarily valued for its energizing properties. The pharmacological effects of Radix Ginseng are attributed to its major bioactive components, known as ginsenoside saponins, of which over 100 distinct types have been identified and isolated from Radix Ginseng [167]. Rb1, Rb2, Rc, Rd, Re, and Rg1 are the most abundant ginsenoside saponins of Radix Ginseng, collectively constituting more than 90% of the total ginsenosides [168]. However, when it comes to cancer treatment, ginsenosides Rg3, Rh2, and compound K are most frequently investigated, exhibiting promising anticancer effects in preclinical studies and even clinical studies [169, 170].

#### Ginsenosides rg3

In numerous preclinical trials, ginsenoside Rg3 has demonstrated a diverse range of biological activities, including, but not limited to, antioxidant, anti-inflammatory, antitumor, and hepatoprotective effects [169, 171]. Rg3 has shown significant clinical benefits when used in combination with TACE for HCC treatment [101]. Furthermore, ginsenoside Rg3 has been validated to exhibit synergistic effects and enhance the sensitivity of cancer cells towards sorafenib and gemcitabine, which are the first-in-line chemotherapeutic agents used for HCC and pancreatic cancer, respectively [172–176]. Mechanistically, the potent antitumor effects of Rg3 mainly derived from its effects on inducing reactive oxygen species (ROS) mediated cancer cell apoptosis [177, 178], and inhibiting VEGF-mediated angiogenesis [179, 180]. Due to the potential combinational effects, Rg3 was also commonly formulated for drug co-delivery with other chemotherapeutics for cancer treatment [181, 182].

#### Ginsenosides rh2

Ginsenosides Rh2 is another bioactive compound of Radix Ginseng with versatile pharmacological activities and excellent medicinal potential. Rh2 was identified to inhibit the proliferation, migration, and invasion of both HCC and pancreatic cancer cells [183, 184]. Additionally, Rh2 also has intrinsic immunomodulatory properties. Li et al. showed that Rh2 significantly reversed the immunosuppressive pancreatic tumor microenvironment by activating dendritic cells via caspase recruitment domain family member 9-BCL10 immune signaling adaptor-MALT1 paracaspase- nuclear factor kappa B (CARD9-BCL10-MALT1/NF-κB) pathway [185]. Likewise, another study illustrated that Rh2 could efficiently potentiated the anti-cancer effect of PD-L1 blockade by promoting CD8^+^ T cell infiltration [186]. Notably, Rh2 demonstrated good biosafety profile and could mitigate doxorubicin-induced cardiotoxicity by targeting inflammatory damages [187].

#### Compound K

Compound K is a ginsenosides-derived bioactive metabolite, which is formed through the metabolic process of ginsenosides by gut microbiota or by enzymatic conversion during the steaming and drying of Radix Ginseng [188]. Compound K has been reported to effectively inhibit glycolysis, which is utilized by cancer cells to meet their high energy demands and support rapid proliferation, namely the Warburg effect [189]. On one hand, Compound K has been demonstrated to enhance the degradation of HIF-1α through ubiquitination and subsequently inhibit the downstream glycolysis pathway [190]. On the other hand, Compound K suppressed Akt/mTOR/c-Myc pathway and key glycolytic enzymes such as Hexokinase 2 (HK2) and pyruvate kinase isozymes M2 (PKM2), thereby thwarting HCC progression [191]. To address the poor water solubility, several delivery systems have been developed to enhance bioavailability and therapeutic efficacy of compound K [192–194].

### Huachansu

Huachansu is derived from the Chinese giant toad and is refined from the lipid-soluble components of dried toad skin [195]. It is primarily used for the treatment of chronic hepatitis B and late-stage tumors. Currently, it is being studied in clinical research for the treatment of advanced malignant tumors such as liver cancer, pancreatic cancer, and lung cancer [195, 196]. Cinobufotalin, bufalin, and bufotalin are considered the main active constituents in Huachansu, and more and more studies have confirmed their anti-tumor effects [195, 197].

#### Bufalin

Bufalin is an endogenous cardiotonic steroid. It is also a bufadienolide toxin found not only in toxic toads but also in many plant or animal species, with potential cardiotonic and anti-tumor activities [198, 199]. It has been extensively studied for its ability to induce cell death, block the cell cycle, and inhibit angiogenesis, among other mechanisms, to inhibit tumor growth and metastasis [199]. Mechanism research showed that Syndecan-4 is directly targeted by bufalin to inhibit cell proliferation, invasion, and angiogenesis in HCC [200]. Study also explored bufalin’s specific mechanisms from the perspective of the tumor microenvironment (TME). Bufalin inhibits the overexpression of the NF-κB p50, promoting the transition of macrophages in TME from the M2 phenotype to the M1 phenotype, thus stimulating the immune response [201]. Additionally, bufalin can improve the efficacy of current cancer therapies by overcoming drug resistance. In a study on drug resistance in pancreatic cancer, the use of cell membrane camouflaged and bufalin-loaded nanoparticles for the treatment of pancreatic cancer revealed that bufalin reverses the drug resistance of pancreatic cancer cells by regulating the nucleotide binding oligomerization domain containing 2/nuclear Factor Kappa B/ATP-binding cassette transporter (NOD2/NF-κB/ABC) signaling pathway [202].

#### Cinobufotalin

Cinobufotalin is a bufadienolide compound derived from toad venom, and it has been extensively studied in lung cancer, nasopharyngeal carcinoma, and HCC [203–205]. Cinobufotalin exerts its anticancer effects through various mechanisms, such as inhibiting cell proliferation and colony formation and blocking cell cycle-related pathways [206]. In the treatment of HCC, cinobufotalin has been found to reduce the mRNA and protein expression of β-catenin, as well as its target genes *MMP7* and dickkopf WNT signaling pathway inhibitor 1 (*DKK1*), which are associated with tumor invasion and metastasis [205]. Research has indeed shown that cinobufotalin induces the expression of enkurin, TRPC channel interacting protein (ENKUR). ENKUR, in turn, acts to antagonize the β-catenin/Jun proto-oncogene, AP-1 transcription factor subunit/myosin heavy chain 9/ubiquitin specific peptidase 7 (β-catenin/c-Jun/MYH9/USP7) pathway, leading to increased degradation of c-Myc ubiquitin and suppresses malignant activities of HCC [207].

### Icaritin

Icaritin is a bioactive compound derived from Folium Epimedii. According to a phase III clinical trial, Icaritin Soft Capsule displayed superior therapeutic efficacy than Huachansu Tablet in patients with advanced HCC. As a result, Icaritin Soft Capsule has been approved by NMPA for the treatment of patients with unresectable HCC who are considered unsuitable candidates for standard treatment options [103, 208]. Preclinical studies have shown that icaritin displays promising therapeutic efficacy in both HCC and cholangiocarcinoma [209, 210].

The antitumor pharmacological action of icaritin is mechanistically rooted in its dual targeting of the cancer cells and the immune system. Icaritin could effectively trigger mitophagy mediated by phosphatase and tensin homolog (PTEN) induced kinase 1 (PINK1)-Parkin signaling through regulating feedforward loop, thereby disrupting mitochondrial homeostasis and HCC growth [211]. Moreover, icaritin could induce HCC cell apoptosis through two mechanisms: direct inhibition of cyclin-dependent kinase 2 (CDK2) and promotion of HMG-box transcription factor 1 (HBP1)-mediated transcriptional repression of alpha-fetoprotein (AFP) [212, 213]. Inspired by the effects of icaritin in inducing mitophagy and apoptosis, Yu et al. combined icaritin with doxorubicin in a codelivery system to trigger potent immunogenic cell death, which further induced robust immunity and significantly repressed HCC progression [214]. In addition, icaritin possesses inherent immunomodulatory properties and effectively counteracts immunosuppression in HCC. Icaritin demonstrated a significant reduction in the expression of PD-L1, a pivotal molecule involved in mediating T cell co-inhibitory signals, not only in HCC cells but also in myeloid-derived suppressor cells (MDSCs) and neutrophils [215, 216]. Mechanistically, icaritin is an inhibitory-κB Kinase alpha (IKK-α) inhibitor, which inhibits the NF-κB signaling pathway by blocking IKK complex formation, and further dose-dependently downregulates PD-L1 [216]. Icaritin was also reported to decrease intratumoral and splenic MDSCs, which are immunosuppressive cells dampening T cell cytotoxicity. By reducing MDSC abundance, icaritin elevated CD8^+^ cytotoxic T cell infiltration, representing a promising adjuvant systemic agent for HCC immunotherapy [217, 218].

### Compound kushen injection

Compound Kushen Injection (CKI) is a Chinese herbal medicine formulation derived from the roots of two medicinal plants, namely Radix Sophorae Flavescentis and Rhizoma Smilacis Glabrae. CKI has shown promising antitumor effects in preclinical study and has been approved by NMPA as single agent or combinational therapeutics for various type of cancer [219]. The clinical combination of CKI with TACE, chemotherapy and radiotherapy in HCC and PCC has been reviewed in Sect. " Clinical evidence and application of traditional Chinese medicine for the treatment of hepatic, biliary, and pancreatic cancer". CKI contains abundant alkaloids, which account for the pharmacological effects exerts by CKI. The primary bioactive alkaloids derived from CKI include oxymatrine, matrine, oxysophocarpine, and sophocarpine, among which oxymatrine and matrine are extensively investigated due to their potent antitumor properties [220].

#### Matrine

Matrine, a natural alkaloid compound from Radix Sophorae Flavescentis, exhibits well-established anticancer activity by inhibiting proliferation, inducing apoptosis, suppressing metastasis, and weakening cancer stemness, making it a promising anticancer small molecule [221]. Zhang et al. suggested that SRC proto-oncogene, non-receptor tyrosine kinase (SRC), which regulates multiple pro-tumor signaling transduction, is the target of matrine. Matrine was proven to inhibit SRC activity by non-competitively blocking the autophosphorylation of Tyr419 within the SRC domain, thereby inhibiting downstream phosphorylation levels of mitogen-activated protein kinase/extracellular signal-regulated kinase 1/2 (MAPK/ERK), Janus kinase 2/signal transducer and activator of transcription 3 (JAK2/STAT3), and PI3K/Akt signaling in cancer cells [222]. In HCC, matrine was revealed to induce caspase-independent program cell death via BH3 interacting domain death agonist (Bid)-mediated nuclear translocation of apoptosis inducing factor [223]. This effect was at least partially related to disruption of intracellular redox balance with increased ROS production caused by matrine. Besides, matrine could inhibit the metastasis of HCC by suppressing the migration and invasion of cancer cells through direct inhibiting MMP9 [224, 225]. Similar to other TCM-derived compounds, matrine exhibits pronounced effects in inducing cell apoptosis in HCC, cholangiocarcinoma, gallbladder cancer, and PCC cells through various mechanisms, including the activation of c-Jun N-terminal kinase-B-cell lymphoma 2/BCL2 like 1-BCL2 associated X, apoptosis regulator/BCL2 antagonist/killer 1 (JNK-Bcl-2/Bcl-xL-Bax/Bak) pathway, suppression of JAK2/STAT3 signaling, inhibition of NF-κB, release of mitochondrial cytochrome c, and activation of caspase-3 [226–229]. Apart from that, Xu et al. found that matrine was able to induce necroptosis in cholangiocarcinoma cells through receptor interacting serine/threonine kinase 3/mixed lineage kinase domain like pseudokinase/reactive oxygen species (RIP3/MLKL/ROS) pathway [230]. Matrine also displayed potent antitumor effects in PCC. Specifically, matrine could serve as an autophagy inhibitor to suppress mitochondrial energy production and thus inhibiting KRAS proto-oncogene, GTPase (KRAS)-driven PCC progression [231]. In recent years, considerable efforts have been made to design and synthesis of novel matrine derivatives with better pharmacokinetics profile and efficacy, suggesting the promising potential of matrine and its derivatives towards clinical translation [232–234].

#### Oxymatrine

Oxymatrine is another alkaloid derived from Sophora flavescens that has been confirmed to possess anticancer, antiviral, and anti-inflammatory properties through numerous studies [235–238]. The associated mechanisms primarily involve inducing apoptosis and inhibiting cell proliferation, as well as affecting various molecular targets involved in cancer progression [239]. Among the identified signaling pathways regulated by this compound, the Akt pathway is one of the most frequently observed [240, 241]. Research on pancreatic cancer has revealed that the anticancer effects of oxymatrine may be attributed to the regulation of the Bcl-2 and inhibitors of apoptosis (IAP) families, the release of mitochondrial cytochrome c, and the activation of caspase-3 [229]. Liver fibrosis is strongly associated with HCC and hepatic stellate cells (HSCs) are a key effector in the progression of liver fibrosis [242]. It was shown that oxymatrine can reduce the secretion of transforming growth factor beta 1 (TGF-β1) by downregulating HMGB1, leading to the inactivation of TGF-β1-mediated HSC activation, thereby effectively alleviating CCl4-induced liver fibrosis [243].

### Kangai injection

Kangai Injection is a well-known Chinese patent medicine used as an adjuvant therapy for various types of cancers in clinical practice. It is formulated by refining three extracts of medicinal herbs, namely Radix Astragali, Radix Ginseng, and oxymatrine [244]. The anticancer effects of ginsenosides and oxymatrine have been mentioned above [242]. In this section we will mainly focus on the specific compounds found in Radix Astragali that possess potential anticancer properties.

#### Astragaloside IV

Astragaloside IV (AS-IV), the main component of Radix Astragali, is a wool wax alcohol-type triterpenoid saponin that has various biological effects, including anti-inflammatory, antidiabetic, and immunomodulatory effects [245–247]. In studies on HCC, AS-IV has been shown to significantly inhibit the development of liver cancer. Researchers have found that AS-IV can suppress HCC through the regulation of the pSmad3C/3L and nuclear factor erythroid 2-related factor 2/Heme Oxygenase 1 (Nrf2/HO-1) pathways [248]. Partial disruption of the TGF-β1/Smad3 signaling pathway by Smad3C attenuates the anti-hepatocarcinogenic effect of AS-IV, while the Nrf2/HO-1 signaling pathway enhances the anti-hepatocarcinogenic effect of AS-IV more effectively [249].

#### Calycosin-7-glucoside

Calycosin-7-glucoside (C7G) is another bioactive compound of Radix Astragali. The inherent anti-inflammatory effects have positioned it as a promising therapeutic candidate for various diseases [250]. With regarding to cancer, C7G could efficiently inhibit the proliferation and trigger the apoptosis of HCC cells. C7G is a potent inhibitor of thioredoxin 1, which plays an important role in regulating cellular redox balance [251] Intervention with thioredoxin 1 by C7G reduced mitochondrial membrane potentialand activated oxidative stress and mitochondria-mediated apoptosis in HCC cells [252, 253]. In a similar study, C7G was reported to induce cell-cycle arrest and apoptosis through ROS-mediated MAPK, STAT3, and NF-κB signaling pathways in HepG2 cells [254]. Intriguingly, C7G was reported to bind to interferon gamma (IFN-γ), causing the folding of the IFN-γ backbone into a more packed structure with higher stability and higher antitumor efficacy in HCC compared with free IFN-γ [255].

### Kanglaite injection

Kanglaite Injection, made from Semen Coicis active ingredient, is a biphasic broad-spectrum anticancer drug that can not only effectively inhibit and kill cancer cells, but also significantly improve immune function. Kanglaite Injection has also been recognized to enhance effect and reduce toxicity of radiotherapy and chemotherapy and possess certain anti-cachexia and analgesic effect on patients with advanced cancer. According to the Pharmacopoeia of the People's Republic of China 2020, triolein is the crucial compound and quality control target of Semen Coicis. The anti-cancer effect against HCC and PCC has been investigated on Kanglaite Injection and Semen Coicis, but not on the monomeric compound triolein.

For HCC, Semen Coicis induced apoptosis in HepG2 cells in a concentration- and time-dependent manner by elevating and prolonging the expression of caspase-8 [256]. In a network pharmacology study, it was speculated that potential mechanisms involved in the anti-cancer Semen Coicis against HCC were enriched for precancerous lesion pathways such as hepatitis B and fatty liver as well as biological pathways such as HIF-1 and TNF [257]. For drug resistant HCC, Kanglaite Injection pretreatment may sensitize HepG2 cells to cisplatin partly by inhibiting the transporter-mediated drug efflux as well as the chemokine like factor (CKLF1)-mediated NF-κB pathway that may contribute to inflammation of tumor microenvironment and chemoresistance [258].

For PCC, Kanglaite Injection could inhibit growth and induce apoptosis in PCC xenografts, possibly by downregulating the expression of phospho-Akt and phospho-mTOR to modulate PI3K/Akt/mTOR pathway [259]. In addition, enhanced PTEN was also found to be involved in Kanglaite injection-induced apoptosis of human PCC cells [260]. Taken together, it could be speculated that Kanglaite Injection may serve as a potential treatment strategy that harbors functional PTEN and inhibiting the PI3K/Akt/mTOR pathway for PCC treatment.

### Xiaochaihu decoction

Xiaochaihu Decoction is a classic TCM prescription widely used for the treatment of chronic hepatitis, acute/chronic cholecystitis, and cholelithiasis, etc. Generally, Xiaochaihu Decoction comprises herbal medicines including Radix Bupleuri, Radix Ginseng, Radix Scutellariae, Rhizoma Pinelliae, Radix Glycyrrhizae, etc., among which Radix Bupleuri serves as the dominant ingredient. Referencing to the Pharmacopoeia of the People's Republic of China 2020, saikosaponin A and saikosaponin D (belonging to triterpenoid saponins) are the key bioactive compounds in Radix Bupleuri, with respective contents of approximately 0.1% and 1.0%, thus listed as the quality control targets. In modern pharmacological studies, saikosaponin A saikosaponin D have been reported with anti-cancer effect against HCC, BTC, and PCC.

#### Saikosaponin A

Saikosaponin A has been found to induce ferroptosis of HCC cells in vitro and in vivo, with increased malondialdehyde (MDA) and iron accumulation and decreased reduced glutathione (GSH), which could be rescued by deferoxamine, ferrostatin-1, and GSH, but not Z-VAD-FMK. Mechanically, saikosaponin A stimulated endoplasmic reticulum stress to upregulate transcription factor 3 (ATF3), followed by inhibition of the expression of cystine transporter solute carrier family 7 member 11 (SLC7A11), which resulted in disordered glutathione metabolic pathway, lipid peroxidation, and finally cell ferroptosis. These findings suggested that the anti-cancer effect of saikosaponin A was mediated by ATF3-dependent cell ferroptosis, which could be a potential target for the treatment of HCC [261].

#### Saikosaponin D

Saikosaponin D has been demonstrated to inhibit proliferation and induce apoptosis of HCC SMMC‑7721 cells by downregulating cyclooxygenase 2 (COX‑2) expression and decreasing prostaglandin E2 production via inhibiting phosphor-signal transducer and activator of transcription 3/hypoxia inducible factor‑1α (p‑STAT3/HIF‑1α) pathway [262]. Another study also revealed that saikosaponin D exerted anti-cancer effect against HCC by suppressing COX-2 via inhibiting p-STAT3/CCAAT enhancer binding protein beta (C/EBPβ) pathway [263]. In addition, saikosaponin D could inhibit HCC development by downregulating expression of syndecan-2, MMP2, MMP13, and tissue inhibitor of metalloproteinase 2 (TIMP-2) in liver of rat with HCC [264]. Moreover, saikosaponin D could increase the radiosensitivity of HCC SMMC-7721 cells by adjusting the G0/G1 and G2/M checkpoints of cell cycle [265].

For pancreatic cancer, saikosaponin D could inhibit proliferation and induce apoptosis of BxPC3, PANC1, and Pan02 cells by triggering cleavage of caspase 3 and caspase 9 and increasing expression of FoxO3a via activating the mitogen-activated protein kinase kinase 4-c-Jun N-terminal kinase (MKK4-JNK) pathway [266]. Saikosaponin D could also inhibit the invasion of pancreatic cancer cells, modulate the immunosuppressive microenvironment, and reactivate the local immune response, by decreasing the shift toward M2 macrophage polarization via downregulating STAT6 phosphorylation and inhibiting PI3K/Akt/mTOR pathway [267]. For intrahepatic cholangiocarcinoma, saikosaponin D could reverse epinephrine- and norepinephrine-induced gemcitabine resistance by controlling glucose metabolism and drug efflux via downregulating β2-adrenergic receptor (ADRB2)/glycolysis pathway [268].

#### Yinchenhao deccotion

Yinchenhao Decoction is also a well-known classic TCM prescription for the treatment of acute icteric hepatitis, cholecystitis, and cholelithiasis, etc. In general, Yinchenhao Decoction consists of several herbal medicines, including Herba Artemisiae Scopariae, Fructus Gardeniae, Radix et Rhizoma Rhei, etc., among which Herba Artemisiae Scopariae act as the principal ingredient. As recommended by the Pharmacopoeia of the People's Republic of China 2020, chlorogenic acid and scoparone (belonging to phenolic acids and coumarins, respectively) are the most essential bioactive compounds of Herba Artemisiae Scopariae, serving as the quality control targets. As reported in the literature, chlorogenic acid and scoparone exhibited anti-cancer effect against HCC and PCC, whereas there is no evidence on their anti-cancer effect against BTC. The secondary medicinal herbal material of Yinchenhao Decoction is Fructus Gardeniae, in which geniposide and genipin are two bioactive compounds with clarified targets in HCC.

#### Chlorogenic acid

For HCC, chlorogenic acid could inhibit the proliferation of HepG2 cells in vitro and the progression of HepG2 xenograft in vivo, with the inactivation of ERK1/2 and downregulation of MMP2 and MMP9 [269]. Chlorogenic acid could also decline the malignant characteristics of HCC cells by inhibiting DNA methyltransferase 1 (DNMT1) expression, which enhanced p53 and p21 activity and resulted in a significant reduction in cell proliferation and metastasis [270]. In addition, chlorogenic acid could sensitize HCC HepG2 and Hep3B cells to 5-fluorouracil treatment by inhibiting the activation of ERK via increasing ROS production [271]. In another study, chlorogenic acid enhanced regorafenib-mediated growth inhibition and apoptosis aggravation in HCC cells, with activation of Annexin V, Bax, and Caspase 3/7 as well as inhibition of Bcl-2 and Bcl-xL, by inhibiting the MAPK and PI3K/Akt/mTORC pathways [272].

For PCC, chlorogenic acid effectively suppressed pancreatic ductal adenocarcinoma cell growth in vitro and in vivo by inducing mitochondrial respiration dysfunction and depressing cellular bioenergetics via modulating the MYC proto-oncogene, bHLH transcription factor-transferrin receptor 1 (c-Myc-TFR1) axis [273]. Chlorogenic acid could also trigger apoptosis and inhibit proliferation, colony formation, migration, and invasion of PANC-28 and PANC-1 cells via the protein kinase B/glycogen synthase kinase 3/ β-catenin (Akt/GSK-3β/β-catenin) pathway, with down-regulated expressions of Akt, p-Akt (Thr308), p-GSK-3β (Ser9), β-catenin, N-cadherin, and vimentin as well as up-regulated expressions of cleaved-caspase 3 and cleaved-caspase 7 [274].

#### Scoparone

Scoparone has been found to promisingly ameliorate the pathological alterations and prevent the development of HCC from nonalcoholic fatty liver disease (NAFLD) in a NAFLD-HCC mouse model by modulating p38 MAPK/Akt/NF-κB signaling cascade, with deactivation of MAPK/Akt pathway and downregulation of NF-κB, TNF-α, monocyte chemoattractant protein-1 (MCP-1), iNOS, COX-2, and MMP9 expressions [275]. Scoparone was also discovered to inhibit pancreatic cancer cell proliferation, migration, and invasion as well as induce cycle arrest and apoptosis in vitro or in vivo through the PI3K/Akt pathway [276].

#### Geniposide and genipin

Geniposide is an iridoid glycoside found in Fructus Gardeniae. It is composed of a genipin molecule bound to a glucose molecule. Geniposide is known for its antioxidant, anti-inflammatory, and neuroprotective effects [277]. Our research discovered that geniposide, a natural compound, functions as an antagonist of TLR4. Through its interaction with TLR4, geniposide effectively inhibits the downstream toll-like receptor 4/MYD88 innate immune signal transduction adaptor (TLR4/MyD88) pathway and suppresses signal transducer and activator of transcription 3/Sp1 transcription factor (STAT3/SP1)-dependent VEGF production. Consequently, geniposide inhibits angiogenesis and hampers the progression of HCC in an orthotopic model [278]. Besides, our recent study showed that genipin, the aglycone of geniposide, also possesses potent antitumor effects. Genipin is a natural antagonist of peroxisome proliferator activated receptor gamma (PPAR-γ), by targeting which genipin induces the degradation of NF-κB p65 and inhibits downstream C–C motif chemokine receptor 2 (CCR2) transcription in macrophages. Thereby, genipin suppresses the postoperative influx of macrophage to the liver and subsequently thwart the recurrence of HCC [279].

## Ongoing clinical trials

To explore the future cancer therapeutic potential of TCM, we summarized the current status of clinical studies investigating TCM-based therapeutics for HCC and PCC (Table 3). Interestingly, there is a notable absence of ongoing trials for BTC, pointing to a potential area for future research. Among the TCM currently under investigation, Huaier Granule stands out as the most extensively studied, with three ongoing multi-center-controlled trials evaluating its combination with TACE for HCC or with chemotherapy for PCC treatment, respectively (registered as NCT05660213, NCT06387368, and NCT06368063, respectively). Besides, icaritin, a single small molecule TCM, is under extensive investigation to assess its preliminary clinical efficacy and safety as combinational adjuvant therapeutics for HCC treatment (registered as NCT05903456 and NCT06285149). In May 2023, a prospective, multi-center, randomized controlled clinical trial was conducted to assess the efficacy of the Qingyi Huaji Optimized Formula, a TCM formulation comprising seven medicinal constituents including Herba Scutellariae Barbatae, Herba Hedyotidis Diffusae, Rhizoma Arisaematis, Fructus Amomi Rotundus, Ganoderma, Herba Gynostemmatis, and Semen Coicis, in patients who were diagnosed with stage IV pancreatic ductal adenocarcinoma and were undergoing gemcitabine treatment (NCT05840341). This formula has displayed significant efficacy in preclinical PCC model [280, 281]. And the trial organizers hypothesized that Qingyi Huaji Optimized Formula may bring clinical benefit for patients by synergizing with standard chemotherapy. These trials may provide insights into the safety, efficacy, and mechanisms of action of TCM formulations in HCC and PCC. The findings from the ongoing trials highlight the growing interest and recognition of TCM's potential in cancer treatment.

**Table 3:** Planned and ongoing clinical study of TCM for the treatment of hepatic, and pancreatic cancer

TCM	Condition	Intervention	Design	Phase	Status	Primary outcome
NCT03515369 Babaodan Oral Capsule	Resectable HCC	Babaodan Oral Capsule + hepatectomy *vs*. Placebo Oral Capsule + hepatectomy	Multicenter, randomized, parallel-controlled, quadruple-blind trial	IV	Unknown	3-year DFS
NCT05152498 Fuzheng Jiedu Huayu Therapy	HBV-associated HCC	Fuzheng Jiedu Huayu Therapy *vs*. routine medical care	Randomized, parallel-controlled, open-label trial	I	Unknown	Time to progression
NCT03356236 Huaier Granule	HCC after local ablation	Huaier Granule *vs*. no treatment	Multicenter, prospective cohort study	NA	Recruiting	96-week PFS
NCT05660213 Huaier Granule	Unresectable HCC	Huaier Granule + atezolizumab + bevacizumab, Huaier Granule + apatinib + camrelizumab, Huaier Granule + sintilimab + bevacizumab *vs*. control	Multicenter, non-randomized, parallel-controlled, open-label trial	IV	Not yet recruiting	One-year ORR
NCT05673824 Huaier Granule	Nephrotoxicity associated with targeted therapy in advanced hepatobiliary malignancies	Huaier Granule + VEGFR- tyrosine kinase inhibitors	Single-arm exploratory study	IV	Recruiting	Effective rate on proteinuria treatment after 8 weeks of Huaier granule
NCT06387368 Huaier Granule	Unresectable PCC	Huaier Granule + capecitabine *vs.* capecitabine	Multicenter, randomized, parallel-controlled, open-label trial	IV	Not yet recruiting	Two-year OS
NCT06368063 Huaier Granule	PCC with radical tumor resection	Huaier Granule *vs.* chemotherapy	Multicenter, randomized, parallel-controlled, open-label trial	IV	Recruiting	Two-year DFS
NCT06285149 Icaritin	Advanced HCC in Child–Pugh B	Icaritin + TACE	Single-arm exploratory study	II	Not yet recruiting	Two-year PFS and ORR
NCT05594927 Icaritin Soft Capsule	Unresectable HCC	Icaritin Soft Capsule *vs*. Huachansu	Multicenter, randomized, parallel-controlled, double-blind, double-dummy trial	III	Recruiting	Two-year OS
NCT05903456 Icaritin Soft Capsule	Unresectable, non-metastatic HCC	Icaritin Soft Capsule + Lenvatinib + TACE	Single-arm, open-label study	II	Not yet recruiting	48-week ORR
NCT04000737 PHY906	HBV-associated HCC	PHY906 + sorafenib *vs*. placebo + sorafenib	Randomized, parallel-controlled, quadruple-blind trial	II	Unknown	Two-year PFS
NCT05840341 Qingyi Huaji Optimized Formula	Advanced PCC	QingyiHuaji optimized formula + standard chemotherapy *vs.* placebo + standard chemotherapy	Multicenter, randomized, parallel-controlled, double-blind trial	III	Recruiting	Two-year OS
NCT04562428 Xiangsha Liujunzi Decoction	Advanced HCC	Xiangsha Liujunzi Decoction *vs*. placebo	Randomized, parallel-controlled, quadruple-blind trial	IV	Unknown	Body weight and quality of life

*DFS* disease-free survival, *OS* overall survival, *PFS* progression-free survival, *ORR* objective response rate

## Discussion

The integration TCM into mainstream cancer management for hepatic, biliary, and pancreatic cancers presents both challenges and promising future directions. This literature review has highlighted the clinical efficacy of several TCM and the potential for TCM-derived lead compounds in anticancer drug discovery. However, several obstacles need to be addressed to facilitate the clinical translation of TCM.

One of the key challenges restricting the globalization of TCM is the regulatory hurdle, which predominantly revolves around the need for standardization and validation of TCM practices in alignment with western medical protocols. Therefore, solid evidences demonstrating the efficacy, safety, and reproducibility of TCM interventions are highly required. While TCM has shown promising results in some clinical study, it remains imperative to conduct further well-designed, multicenter large cohort, as well as placebo-double-blinded randomized controlled trials to validate the efficacy of TCM as either monotherapy or combinational agents. Additionally, concerns arise regarding prolonged safe use and potential interactions with standard treatments over long periods, especially considering that most TCM requires long-term continuous administration. TCM with stringent standardization typically features a well-defined safe daily dosage. And botanical nature makes it tend to have intrinsic better biocompatibility. Nonetheless, comprehensive pharmacovigilance is still imperative to monitor potential herb-drug interactions, cumulative toxicity, and adverse effects, especially for patients with impaired hepatobiliary functions. Future phase I/II trials can be further extended to a long period investigating the prolonged safe use and long-term efficacy of TCM.

Standardization and quality control of TCM products also pose challenges. TCM treatments often involve complex herbal formulations, making it crucial to ensure consistent quality, purity, and potency of these products. Developing standardized protocols for TCM preparations and establishing quality control measures will not only enhance treatment effectiveness but also facilitate regulatory approval and acceptance by the medical community [282]. Cultural barriers also pose significant hurdles, rooted in the fundamental differences between TCM philosophy and western medical paradigms. The divergent approaches to disease conceptualization, diagnostic methodologies, and perceptions of evidence-based medicine hinder the integration of TCM into western oncology practices.

TCM contains a plethora of bioactive compounds with potential anti-tumor properties, presenting a valuable resource for anti-drug discovery. Moreover, identification of effective bioactive compounds from complex formulas bypasses the intricate process of TCM standardization and quality control. Employing a classical rational drug discovery and design approach, this strategy holds the potential to advance the credibility, accessibility and internationalization of TCM, as exemplified by successful translation of compounds such as artemisinin, icaritin, and paclitaxel [283]. The application of advanced technologies in the purification and screening of bioactive compounds from TCM has transformed the drug discovery landscape. These advanced technological tools, encompassing a spectrum of methodologies such as high-resolution mass spectrometry, high-resolution MS/MS database, and high-throughput screening platforms, have significantly enhanced the efficiency and precision of isolating and characterizing bioactive constituents from complex TCM formulations [284]. Additionally, artificial intelligence (AI) also aids in drug discovery from TCM by efficiently analyzing vast datasets of bioactive compounds, predicting interactions with cancer targets, and accelerating the identification of potential drug candidates. Through virtual screening, predictive modeling, and personalized medicine approaches, AI in the future may substantially expedite the protracted process of drug discovery [7]. However, it is important to acknowledge that unmodified natural products may exhibit suboptimal efficacy or encounter challenges related to absorption, distribution, metabolism, excretion, and toxicity. To address these limitations and progress natural product hits into drug leads, medicinal chemical modifications are often necessary. Through the refinement of chemical structures, researchers can optimize the pharmacokinetics and pharmacodynamics properties of these compounds, increasing their therapeutic potential and guiding them towards clinical trials and successful drug development. Drug discovery from TCM emphasizes the need for collaborative efforts between traditional medicine practitioners and modern scientists to harness the full therapeutic potential of TCM in combating cancer effectively.

Moreover, the integration of TCM with conventional cancer treatments requires a multidisciplinary approach. The identification of specific biomarkers has the potential to tailor the rational and effective utilization of TCM in clinical setting by offering insights into patient responses and directing personalized therapy [285]. Besides, collaboration among healthcare professionals, including oncologists, TCM practitioners, pharmacists, and researchers, is essential to develop comprehensive treatment plans that optimize patient outcomes. This collaboration should also extend to the design of clinical trials, where TCM interventions can be incorporated into standard protocols to evaluate their optimal timing of administration and synergistic effects, especially considering that currently most TCM is combined with conventional therapy.

In conclusion, the clinical integration of TCM for hepatic, biliary, and pancreatic cancer management presents challenges related to scientific validation, standardization, and multidisciplinary collaboration. Addressing these challenges through rigorous research, quality control measures, and collaborative efforts will pave the way for the effective integration of TCM into mainstream cancer care.

## Supplementary Information

Supplementary Material 1.

## Data Availability

This published article and its supplementary information files include all data generated or analyzed during this study.

## Abbreviations

TCM: Traditional Chinese medicine
RCT: Randomized clinical trials
NMA: Network meta-analysis
HCC: Hepatocellular carcinoma
TACE: Transarterial chemoembolization
TKI: Tyrosine kinase inhibitors
PD-1: Programmed cell death protein 1
HR: Hazard ratio
NMPA: National Medical Products Administration
OS: Overall survival
DFS: Disease-free survival
PFS: Progression-free survival
RFS: Recurrence-free survival
RR: Relative risk
OR: Odds ratio
QoL: Quality of life
CI: Confidence interval
BTC: Biliary tract cancer
GBC: Gallbladder cancer
ICC: Intrahepatic cholangiocarcinoma
ECC: Extrahepatic cholangiocarcinoma
SCRT: Stereotactic body radiotherapy
KPS: Karnofsky performance status
PCC: Pancreatic cancer
TTP: Time-to-progression
NO: Nitric oxide
TNF-α: Tumor necrosis factor α
IL-6: Interleukin-6
TLR4: Toll-like receptor
NF-κB: Nuclear factor kappa B
MAPK: Mitogen-activated protein kinase
EMT: Epithelial-mesenchymal transition
HIF-1α: Hypoxia inducible factor 1 subunit alpha
VEGF: Vascular endothelial growth factor
MTDH: Metadherin
MMPs: Matrix metalloproteases
PI3K/Akt/mTOR: Phosphoinositide 3-kinase/protein kinase B/mammalian target of rapamycin
CDK9: Cyclin-dependent kinase 9
HMGB1: High mobility group box 1
BCL2: B-cell lymphoma 2
HK2: Hexokinase 2
PKM2: Pyruvate kinase isozymes M2
TME: Tumor microenvironment
PTEN: Phosphatase and tensin homolog
AFP: Alpha-fetoprotein
MDSC: Myeloid-derived suppressor cells
IKK-α: Inhibitory-κB Kinase alpha
NRF2: Nuclear factor erythroid 2-related factor 2
CKI: Compound Kushen Injection
ERK: Extracellular signal-regulated kinase
TGF-β1: Transforming growth factor beta 1
JAL2/STAT3: Janus kinase 2/signal transducer and activator of transcription 3
IFN-γ: Interferon gamma
ROS: Reactive oxygen species
GSH: Glutathione
COX2: Cyclooxygenase 2
TIMP2: Tissue inhibitor of metalloproteinase 2
NAFLD: Nonalcoholic fatty liver disease
PPAR-γ: Peroxisome proliferator activated receptor gamma
AI: Artificial intelligence

## Data availability

No datasets were generated or analysed during the current study.
