# Supplementary material for: Traditional Chinese medicine for the treatment of cancers of hepatobiliary system: from clinical evidence to drug discovery
Source: Mol Cancer. 2024 Oct 1;23:218. doi: 10.1186/s12943-024-02136-2 (PMC11443773; doi:10.1186/s12943-024-02136-2)
Supplement: Supplementary file 1 — Supplementary Material 1. [file 12943_2024_2136_MOESM1_ESM.docx]

**SI-Table 1**. Original resource of medicinal constituent of traditional Chinese medicine for the treatment of hepatic, biliary, and pancreatic cancer

| **Chinese name** | **Pharmaceutical name** | **Original resource** |
| --- | --- | --- |
| Bai-Hua-She-She-Cao | Herba Hedyotidis Diffusae | *Hedyotis diffusa* Willd.  *Hedyotis corymbosa* (L.) Lamk. |
| Bai-Shao | Radix Paeniae Alba | *Paeonia lactiflora* Pall. |
| Bai-Tu-Fu-Ling | Rhizoma Heterosmilacis | *Heterosmilax japonica* Kunth |
| Bai-Zhu | Rhizoma Atractylodis Macrocephalae | *Atractylodes macrocephala* Koidz. |
| Bai-Zhu (Xian) | Rhizoma Atractylodis Macrocephalae (Recens) | *Atractylodes macrocephala* Koidz. |
| Ban-Lan-Gen | Radix Isatidis | *Isatis indigotica* Fort. |
| Ban-Mao | Mylabris | *Mylabris phalerata* Pallas  *Mylabris cichorii* Linnaeus |
| Ban-Xia | Rhizoma Pinelliae | *Pinellia ternata* (Thunb.) Breit. |
| Ban-Zhi-Lian | Herba Scutellariae Barbatae | *Scutellaria barbata* D. Don |
| Bie-Jia | Carapax Trionycis | *Trionyx sinensis* Wiegmann |
| Bing-Pian | Borneolum Syntheticum | Borneolum Syntheticum |
| Chai-Hu | Radix Bupleuri | *Bupleurum chinense* DC.  *Bupleurum scorzonerifolium* Willd. |
| Chan-Su | Venenum Bufonis | *Bufo bufo gargarizans* Cantor  *Bufo melanostictus* Schneider |
| Chen-Pi | Pericarpium Citri Reticulatae | *Citrus reticulata* Blanco  *Citrus reticulata* 'Chachi'  *Citrus reticulata* 'Dahongpao'  *Citrus reticulata* 'Unshiu'  *Citrus reticulata* 'Tangerina' |
| Chi-Shao | Radix Paeoniae Rubra | *Paeonia lactiflora* Pall. |
| Chuan-Xiong | Rhizoma Chuanxiong | *Ligusticum chuanxiong* Hort. |
| Chui-Pen-Cao | Herba Sedi | *Sedum sarmentosum* Bunge |
| Ci-Wu-Jia | Radix et Rhizoma seu Caulis Acanthopanacis Senticosi | *Acanthopanax senticosus* (Rupr. et Maxim.) Harms |
| Da-Huang | Radix et Rhizoma Rhei | *Rheum palmatum* L.  *Rheum tanguticum* Maxim. ex Bal£.  *Rheum officinale* Baill. |
| Da-Zao | Fructus Jujubae | *Ziziphus jujuba* Mill. |
| Dan-Shen | Radix Salviae Miltiorrhizae | *Salvia miltiorrhiza* Bunge. |
| Dang-Gui | Radix Angelicae Sinensis | *Angelica sinensis* (Oliv.) Diels |
| Dang-Shen | Radix Codonopsis | *Codonopsis pilosula* (Franch.) Nannf.  *Codonopsis pilosula* Nannf. var. *modesta* (Nannf.) L. T. Shen  *Codonopsis tangshen* Oliv. |
| Dong-Chong-Xia-Cao | Cordyceps | *Cordyceps sinensis* (BerK.) Sacc. |
| Dou-Kou | Fructus Amomi Rotundus | *Amomum kravanh* Pierre ex Gagnep.  *Amomum compactum* Soland ex Matan |
| E-Zhu | Rhizoma Curcumae | *Curcuma phaeocaulis* Val.  *Curcuma Kwangsiensis* S. G.Lee et C. F. Liang  *Curcuma wenyujin* Y.H. Chen et C. Ling |
| Fa-Ban-Xia | Rhizoma Pinelliae Praeparatum | *Pinellia ternata* (Thunb.) Breit. |
| Feng-Fang | Nidus Vespae | *Polistes olivaceous* (DeGeer)  *Polistes japonicus* Saussure  *Parapolybia varia* Fabricius |
| Fo-Shou | Fructus Citri Sarcodactylis | *Citrus medica* L. var. *sarcodactylis* Swingle |
| Fu-Ling | Poria | *Poria cocos* (Schw.) Wolf |
| Gan-Cao | Radix Glycyrrhizae | *Glycyrrhiza uralensis* Fisch.  *Glycyrrhiza inflata* Bat.  *Glycyrrhiza glabra* L. |
| Hong-Hua | Flos Carthami | *Carthamus tinctorius* L. |
| Huai-Er | Poria Robiniophila | *Poria robiniophila* (Murrill) Ginns  (formerly *Trametes robiniophila* Murr) |
| Huang-Qi | Radix Astragali | *Astragalus membranaceus* (Fisch.) Bge. var. *mongholicus* (Bge.) Hsiao  *Astragalus membranaceus* (Fisch.) Bge. |
| Huang-Qi (Xian) | Radix Astragali (Recens) | *Astragalus membranaceus* (Fisch.) Bge. var. *mongholicus* (Bge.) Hsiao  *Astragalus membranaceus* (Fisch.) Bge. |
| Huang-Qin | Radix Scutellariae | *Scutellaria baicalensis* Georgi |
| Ji-Nei-Jin | Endothelium Corneum Galli Gigerii | *Gallus gallus domesticus* Brisson |
| Jiang-Can | Bombyx Batryticatus | *Bombyx mori* Linnaeus (The larva infected by *Beauveria bassiana* (Bals.) Vuillant) |
| Jiang-Huang | Rhizoma Curcumae Longae | *Curcuma longa* L. |
| Jiao-Gu-Lan | Herba Gynostemmatis | *Gynostemma pentaphyllum* (Thunb.) Makino |
| Jin-Qian-Bai-Hua-She (Xian) | Parvus Bungarus (Recens) | *Bungarus multicinctus* Blyth |
| Jin-Qian-Cao | Herba Lysimachiae | *Lysimachia christinae* Hance |
| Ku-Shen | Radix Sophorae Flavescentis | *Sophora flavescens* Ait. |
| Lian-Qiao | Fructus Forsythiae | *Forsythia suspensa* (Thunb.) Vahl |
| Ling-Xiao-Hua | Flos Campsis | *Campsis grandiflora* (Thunb.) K. Schum.  *Campsis radicans* (L.) Seem. |
| Ling-Zhi | Ganoderma | *Ganoderma lucidum* (Leyss. ex Fr.) Karst.  *Ganoderma sinense* Zhao, Xu et Zhang |
| Long-Dan | Radix et Rhizoma Gentianae | *Gentiana manshurica* Kitag.  *Gentiana scabra* Bunge  *Gentiana triflora* pall  *Gentiana rigescens* Franch. |
| Long-Kui | Herba Solani Nigri | *Solanum nigrum* L. |
| Ma-Qian-Zi | Semen Strychni | *Strychnos nux-vomica* L. |
| Mai-Dong | Radix Ophiopogonis | *Ophiopogon japonicus* (L. f) Ker-Gawl. |
| Mao-Ren-Shen | Radix Actinidiae Valvatas | *Actinidia valvata* Dunn |
| Mu-Dan-Pi | Cortex Moutan | *Paeonia suffruticosa* Andr. |
| Mu-Li (Xian) | Concha Ostreae (Recens) | *Ostrea gigas* Thunberg  *Ostrea talienwhanensis* Crosse  *Ostrea rivularis* Gould |
| Nan-Sha-Shen | Radix Adenophorae | *Adinophora stricta* Miq.  *Adenophora tetraphylla* (Thunb.) Fisch. |
| Nv-Zhen-Zi | Fructus Ligustri Lucidi | *Ligustrum lucidum* Ait. |
| Qi-She (Xian) | Agkistrodon (Recens) | *Agkistrodon acutus* (Guenther) |
| Qi-Ye-Yi-Zhi-Hua | Rhizoma Paridis Chinensis | *Paris polyphylla* Smith var. *chinensis* (Franch.) Hara |
| Qing-Ban-Xia | Rhizoma Pinelliae Praeparatum cum Alumine | *Pinellia ternata* (Thunb.) Breit. |
| Ren-Gong-Niu-Huang | Calculus Bovis Artifactus | Pulvis Billis Bovis, bilirubin, hyodesoxycholic acid, cholalic acid, taurine, cholesterol, and microelement |
| Ren-Shen | Radix Ginseng | *Panax ginseng* C. A. Mey. |
| San-Qi | Radix et Rhizoma Notoginseng | *Panax notoginseng* (Burk.) F. H. Chen |
| Shan-Ci-Gu | Pseudobulbus Cremastrae  Pseudobulbus Pleiones | *Cremastra appendiculata* (D.Don) Makino  *Pleione bulbocodioides* (Franch.) Rolfe  *Pleione yunnanensis* Rolfe |
| Shan-Yao | Rhizoma Dioscoreae | *Dioscorea opposita* Thunb. |
| She-Liu-Gu | Rhizoma Amcrphophalli | *Amorphophallus konjac* K. Koch  *Amorphophallus rivieri* Durieu  *Amorphophallus dunnii* Tutch.  *Amorphophallus sinensis* Belval  *Amorphophallus variabilis* BI.  *Amorphophallus mairei* Levl. |
| Sheng-Jiang | Rhizoma Zingiberis Recens | *Zingiber officinale* Roscoe |
| Shou-Gong (Xian) | Gecko House Lizard (Recens) | *Gekko subpalmatus* Gunther  *Gekko swinboana* Gunther  *Gekko swinhonis* Guenther  *Gekko japonicus* (Dumeril et Bibron) |
| Shu-Di-Huang | Radix Rehmanniae Preparata | *Rehmannia glutinosa* Libosch. |
| Shui-Hong-Hua-Zi | Fructus Polygoni Orientalis | *Polygonum orientale* L. |
| Shui-Zhi | Hirudo | *Hirudo nipponica* Whitman  *Whitmania pigra* Whitman  *Whitmania acranulata* Whitman |
| Tao-Ren | Semen Persicae | *Prunus persica* (L.) Batsch  *Prunus davidiana* (Carr.) Franch. |
| Teng-Li-Gen | Radix Actinidiae | *Actinidia chinensis* Planch. |
| Tu-Fu-Ling | Rhizoma Smilacis Glabrae | *Smilax glabra* Roxb. |
| Wu-Ling-Zhi | Faeces Trogopterori | *Trogopterus xanthippes* Milne-Edwards |
| Wu-Shan-Yin-Yang-Huo | Folium Epimedii Wushanensis | *Epimedium wushanense* T. S. Ying |
| Wu-Yao | Radix Linderae | *Lindera aggregata* (Sims) Kosterm. |
| Xiang-Fu | Rhizoma Cyperi | *Cyperus rotundus* L. |
| Ya-Dan-Zi | Fructus Bruceae | *Brucea javanica* (L.) Merr. |
| Yan-Hu-Suo | Rhizoma Corydalis | *Corydalis yanhusuo* W. T. Wang |
| Yang-Tai-Pan | Emoryp Caprae seu Ovis | *Capra hircus* Linnaeus  *Ovis aries* Linnaeus |
| Yi-Yi-Ren | Semen Coicis | *Coix lacryma-jobi* L. var. *ma-yuen* (Roman.) Stapf |
| Yi-Yi-Ren (Xian) | Semen Coicis (Recens) | *Coix lacryma-jobi* L. var. *ma-yuen* (Roman.) Stapf |
| Yin-Chen | Herba Artemisiae Scopariae | *Artemisia scoparia* Waldst. et Kit.  *Artemisia capillaris* Thunb. |
| Yin-Yang-Huo | Folium Epimedii | *Epimedium brevicornum* Maxim.  *Epimedium sagittatum* (Sieb.et Zucc.) Maxim.  *Epimedium pubescens* Maxim.  *Epimedium koreanum* Nakai |
| Yu-Jin | Radix Curcumae | *Curcuma wenyujin* Y. H. Chen et C. Ling  *Curcuma longa* L.  *Curcuma kwangsiensis* S. G. Lee et C. F. Liang  *Curcuma phaeocaulis* Val. |
| Zhi-Qiao | Fructus Aurantii | *Citrus aurantium* L.  *Citrus aurantium* 'Huangpi'  *Citrus aurantium* 'Daidai'  *Citrus aurantium* 'Chuluan'  *Citrus aurantium* 'Tangcheng' |
| Zhi-Zi | Fructus Gardeniae | *Gardenia jasminoides* Ellis |
| Zi-Shen | Herba Salviae Chinesnsis | *Salvia chinensis* Benth. |
